# Primary mouse myoblast metabotropic purinoceptor profiles and calcium signalling differ with their muscle origin and are altered in mdx dystrophinopathy

**DOI:** 10.1038/s41598-023-36545-y

**Published:** 2023-06-08

**Authors:** Justyna Róg, Aleksandra Oksiejuk, Dariusz C. Górecki, Krzysztof Zabłocki

**Affiliations:** 1 Laboratory of Cellular Metabolism, Nencki Institute of Experimental Biology Polish Academy of Sciences, Warsaw, Poland; 2 School of Pharmacy and Biomedical Sciences, University of Portsmouth, St Michael’s Building, White Swan Road, Portsmouth, PO1 2DT UK

**Keywords:** Biochemistry, Biological techniques, Cell biology

## Abstract

Mortality of Duchenne Muscular Dystrophy (DMD) is a consequence of progressive wasting of skeletal and cardiac muscle, where dystrophinopathy affects not only muscle fibres but also myogenic cells. Elevated activity of P2X7 receptors and increased store-operated calcium entry have been identified in myoblasts from the mdx mouse model of DMD. Moreover, in immortalized mdx myoblasts, increased metabotropic purinergic receptor response was found. Here, to exclude any potential effects of cell immortalization, we investigated the metabotropic response in primary mdx and wild-type myoblasts. Overall, analyses of receptor transcript and protein levels, antagonist sensitivity, and cellular localization in these primary myoblasts confirmed the previous data from immortalised cells. However, we identified significant differences in the pattern of expression and activity of P2Y receptors and the levels of the “calcium signalling toolkit” proteins between mdx and wild-type myoblasts isolated from different muscles. These results not only extend the earlier findings on the phenotypic effects of dystrophinopathy in undifferentiated muscle but, importantly, also reveal that these changes are muscle type-dependent and endure in isolated cells. This muscle-specific cellular impact of DMD may not be limited to the purinergic abnormality in mice and needs to be taken into consideration in human studies.

## Introduction

Duchenne muscular dystrophy (DMD) is an inherited neuromuscular disease, which results in severe disability and premature death due to respiratory or cardiac failure^1^. Moreover, DMD affects the nervous system leading to neuropsychiatric symptoms. DMD is a consequence of out-of-frame mutations in the *DMD* gene located on chromosome X and encoding dystrophins. Thus, this disease almost exclusively affects boys, with a prevalence of 1 per 5000 live births^2^. The *DMD* gene is composed of 79 exons and, in humans, has eight known promoters. Four of them, located at the 5′ end of the gene, drive expression of three 427 kDa full-length dystrophins, with only subtle differences in their N-termini, but expressed in a tissue-specific manner, and a 412 kDa embryonic isoform^3^. The remaining four, progressively shorter, dystrophins are expressed in various tissues and play specific roles, but none of them can functionally replace the 427 kDa isoforms. Thus, loss of full-length dystrophin is necessary and sufficient to cause DMD. In myofibres, 427 kDa dystrophin is a cytoskeletal protein, which tethers intracellular actin filaments with the dystrophin-associated protein (DAP) complex located in the sarcolemma. Further indirect interactions involve specific intra- and extracellular proteins^4^.

The lack of dystrophin is thought to prevent proper DAP formation, which is a scaffold and an important element of the cellular signalling network^5^. Our current understanding of the role of dystrophin assumes the stabilization of the sarcolemma and ultimately of muscle fibres during contraction. However, such a mechanism does not explain abnormalities found in non-contracting dystrophic cells, such as neurons^6^, satellite cells and myoblasts^7–9^, lymphocytes^10^, endotheliocytes^11^, platelets^12,13^, thymic cells^14^, and across a spectrum of normal epithelial tissues at the level of typical housekeeping genes^15^. Indeed, a growing body of data shows that dystrophin's “mechanical” role does not explain all of the cellular consequences of DMD. Particularly, dystrophy-related alterations in cells that seem not to produce full-length dystrophins are not easily explained [Ref.^10^, for rev. see Ref.^16^].

Myoblasts, undifferentiated, single-nucleated, proliferating muscle cells do not contain 427 kDa dystrophin detectable by standard methods such as Western blotting. However, dystrophic human and mouse myoblasts exhibit profound transcriptomic and functional alterations^9^ and present with many phenotypic consequences of *DMD* mutations^8,17–20^. The molecular mechanism(s) behind this phenomenon are not completely understood^9^, hence the need for further studies. In the course of DMD, regeneration may partially counteract muscle wasting, but it is not clear whether dystrophic myogenic cells can fully support regenerative mechanisms^9,21,22^. Indeed, muscle regeneration recapitulates, to a large extent, muscle development and there is increasing evidence that DMD impairs early ontogenesis^23^. The multistep regeneration process begins when satellite cells are activated to divide, with resulting myoblasts proliferating and migrating to the site of damage, fusing and differentiating into myotubes and, eventually, mature muscle fibres.

Variations in the intracellular calcium that are reflected by changes in the global or local Ca^2+^ concentrations are of crucial importance for muscle differentiation to occur^24^. Calcium signalling is activated, modulated and terminated by orchestrated action of a spectrum of molecular tools (“calcium toolkit”), which maintain the appropriate distribution of Ca^2+^ and allow calcium cations to flow up and down their concentration gradients in a very precisely controlled manner^25^. Importantly, calcium dyshomeostasis is a hallmark of dystrophinopathy, being found across all affected cells^16^. Purinoceptors have been identified as important in this process^26,27^. These receptors are activated by extracellular nucleotides (ATP in particular) and initiate specific cellular calcium signals. The ionotropic P2X receptors function as ATP-gated Ca^2+^ channels in the plasma membrane, while the P2Y metabotropic receptor signalling involves protein G and phospholipase-mediated calcium release from intracellular stores. Previously, we and others have provided evidence of the existence of the purinergic phenotype involving P2RX7 receptors in mdx mouse muscles, the most commonly used animal model of DMD, where ablation of pharmacological inhibition of this receptor alleviated the dystrophic pathology^28–30^. Using immortalized mdx myoblasts, we also demonstrated substantial differences concerning metabotropic purinergic responses, store-operated calcium entry, and specific proteins involved in the cellular calcium signalling pathways^17,20,31,32^. We also identified mdx-related alterations in mitochondrial network organization and cellular energy metabolism^18^.

Immortalized w/t and mdx myoblasts have been derived from the immorto mice^33^, and therefore are identical in their genetic makeup except for the mdx mutation. They have also grown under identical conditions for a long time. This allows the identification of differences reflecting intrinsic cellular consequences of DMD, not influenced by the cell niche (inflammatory in mdx), isolation procedures or different growth conditions. Moreover, primary myoblasts isolated from normal and dystrophic muscles may not only exhibit differences resulting from the different muscle environments from which they are derived, but also tend to differentiate spontaneously at different rates^8^, while immortalized cells maintain their myoblast status under the designed growth conditions. However, after the long-term culture, immortalized myoblasts acquire changes resulting from the transcriptomic drift and adaptation to artificial culture conditions, which need to be separated from dystrophic alterations. N.B., despite all these adaptations, the dystrophic phenotype remains^9^. Yet, cell lines do not allow identifying variability between myoblasts derived from specific muscles. It is well known that different muscles respond differently to the loss of dystrophin^34^ and that muscle cells cultured in vitro remember their in vivo origins^35,36^. Thus, analyses in both established and primary cells will deliver more complete and compatible data.

Here, we investigated the expression and activity of metabotropic receptors in primary myoblasts obtained from four different w/t and mdx leg muscles. Moreover, in myoblasts derived from specific muscles, we compared the distribution of key proteins involved in cellular calcium homeostasis that were previously found altered in the dystrophic myoblast cell line^32^. Notably, the consequences of the mdx mutation exhibited muscle-specific patterns and the genotype–phenotype correlations differed in myoblasts isolated from distinct muscles, as these cells seem to retain their specific characteristics upon isolation. Therefore, previous results, obtained using cell lines or primary myoblasts isolated from a single muscle should not be simply translated to all myoblasts, irrespective of their muscle origin, even though all dystrophic cells consistently display the general alteration, which is aberrant calcium signalling.

## Material and methods

### Primary myoblasts isolation and culture

The mdx mouse (C57BL/10ScSn-Dmd/J) does not express the full-length dystrophins due to a nonsense mutation in exon 23; therefore, it is a widely used model for human DMD. All animals used in the experiments were kept at 20–24 °C, humidity 45–65% and a 12-h light–dark cycle. All efforts were taken to minimize the number of animals used and reduce the stress placed upon them. All experiments had the approval of the Animal Welfare Committee at Nencki Institute of Experimental Biology in Warsaw, according to Directive 2010/63/EU of the European Parliament and of the Council of 22 September 2010 on the protection of animals used for scientific purposes (incl. annexe IV). Mice were killed by isoflurane inhalation followed by cervical dislocation. The study is reported in accordance with ARRIVE guidelines (https://arriveguidelines.org). Myoblasts were isolated from the hindlimb muscle of mdx and control (C57BL/10ScSnJ) 8-week-old male mice (Jackson Laboratory, Bar Harbor, Main, USA). Isolation and purification of satellite cells from Tibialis anterior (TA), Gastrocnemius (GC), Soleus (SOL) and Flexor Digitorum Brevis (FDB) muscles and myoblast culture procedures were performed as described^37^. Briefly, whole muscles were sterilized in PBS with betadine and then digested with 0.2% collagenase type IV in DMEM for 1.5 h at 37 °C, rinsed in DMEM supplemented with 1 g/l glucose, 1 mM pyruvate, 4 mM l-glutamine, 10% HS, 0.5% chicken embryo extract (CEE), 1000 U/l penicillin (1000 U/l) and 1 mg/l streptomycin and triturated with pipettes of gradually decreasing diameter. Entire fibres were separated, rinsed four times with the same medium and finally transferred into DMEM (composition as above, supplemented with 20% FBS). Purified fibres were dispersed by forcing through 18-gauge injection needles, filtered through 40 µm pore diameter nylon bolting cloth, put into collagen-coated culture dishes and incubated in the same medium at 37 °C in a humidified atmosphere of air (95%) and CO_2_ (5%) for 3–5 days. Then, the emergent myogenic cells were passaged and plated into collagen-coated dishes. Tests were performed on cells 48 h after plating at the confluency appropriate for each experiment (see below), but identical for both genotypes. The purity of myoblast culture was confirmed by the enumeration of cells stained for the Myogenic Determination Gene MyoD (see Supplementary 1). As only myoblasts express this myogenic factor, the percentage of positively-stained cells informed on the culture homogeneity (Supplementary Data 1).

### RNA extraction, reverse transcription and quantitative RT-PCR

Total RNA was isolated from cells (70% confluency) using the Trizol method (TRI reagent, T9424; Sigma). The quality and quantity of samples were determined using a NanoDrop spectrophotometer. Only RNA with an absorbance ratio of 260/280 between 1.8 and 2.0 was used for reverse transcription. Complementary DNA (cDNA) was synthesized from 2 μg of total RNA using First Strand cDNA Synthesis Kit with M-MLV reverse transcriptase and oligo(dT) primers (#K1612, Thermo Fischer Scientific), according to the manufacturer's instructions. RT-qPCR was performed using TaqMan Fast Universal PCR Master Mix (4,352,042, Applied Biosystems) and TaqMan Gene Expression Assays (the primers ID: Mm00435471_m1 for p2ry1, Mm01274119_m1 for p2ry2, Mm00445136_s1 for p2ry4 and Mm01275472_m1 for p2ry6, Mm00446026_m1 for p2ry12 and Mm00546978_m1 for p2ry13) on the 7500 ABI Prism Real-Time PCR System (Applied Biosystems). The level of expression of target genes was normalised to the expression of the GAPDH housekeeping gene (primer ID: Mm99999915_g1) previously found to be stable across myoblasts from different genotypes^8^. The relative gene expression was determined by the 2^−ΔΔ^Ct method using StepOne Software.

### Cell lysis, protein electrophoresis and analysis

Proteins were extracted from adherent cells (70% confluency) by scraping them into the extraction buffer (1 × LysisM, 1 × protease inhibitor cocktail, 2 × phosphatase inhibitor cocktail [all Roche], 2 mM sodium orthovanadate [Sigma]), dispersing with pipetting followed by repeated extrusion through the syringe needle (0.5 mm in diameter) and incubation of the suspension on ice for 20 min. After centrifugation (15,000×*g*, for 20 min at 4 °C), protein concentrations in supernatants were determined using the Bradford assay (Bio-Rad). The proteins (20 μg per each sample) were mixed with the sample buffer at a 3:1 v/v ratio, heated for 5 min at 95 °C, chilled on ice and either stored at − 80 °C or immediately separated on 0.1% SDS polyacrylamide gels (6–12% w/v, depending on the molecular mass of proteins of interest) and electroblotted onto Immobilon-PVDF Transfer Membrane (Merck Millipore). Blots were blocked in 5% w/v non-fat milk or 5% BSA (Albumin, Bovine Serum, 12659, Merck Millipore) powder solved in 1 × TBS-T, 0.01% v /v Tween-20 (Sigma) for 1 h at room temperature (RT) before probing with appropriate primary antibody diluted in a blocking buffer containing 2.5% milk or 5% BSA, depending on the antibody, incubated overnight at 4 °C, with agitation. The following primary antibodies were used: P2RY1 (1:270, APR-009), P2RY2 (1:270, APR-010), P2RY4 (1:300, APR-006), P2RY6 (1:250, APR-011), P2RY12 (1:270, APR-012), P2RY13 (1:270, APR-017); all Alomone Labs, calsequestrin (ab126241), calreticulin (ab128885), SERCA1 (ab124501) and SERCA2 (ab91032); all Abcam, diluted 1:1000, Gαq11 (06-709 Merck Millipore, 1:1000,), PLCβ isoforms 3–4 (sc-133231, sc-166131, respectively; Santa Cruz Biotechnology, all diluted 1:100,), NCX1 (R3F1 Swant, 1:1000), NCX3 (ab84708 Abcam, 1:500), PMCA (ab2825 Abcam, 1:1000) and IP3R (#8568 Cell Signalling Technology, 1:1000). All dilutions were made in BSA solution as described above,). Membranes were washed (3 ×) with 1 × TBS-T for 10 min each wash and incubated with anti-Rabbit (ab6721, Abcam, 1:5000,) or anti-Mouse (ab6728, Abcam, 1:3000) horseradish peroxidase-conjugated secondary antibody for 1 h at RT. Specific protein bands were visualized using the luminol-based substrate (Millipore) and imaged using Fusion FX (Vilber Lourmat) instrument. β-tubulin (ab21058 Abcam 1:10000) antibody was used to correct for protein-loading. Densitometric analyses of specific protein bands were made using exposure times within the linear range and the integrated density measurement function of BIO-1D (Vilber Lourmat).

### [Ca^2+^]c measurements in myoblasts

Myoblasts were cultured on glass coverslips in 35 mm diameter dishes for 48 h under the conditions described above. Cells (70–80% confluent) were loaded with 2 μM Fura 2 AM (MolecularProbes, Oregon) in the serum-free culture medium for 20 min at 37 °C in a humidified atmosphere of 95% O_2_ and 5% CO_2_. The cells were then washed twice with the solution composed of 5 mM KCl, 1 mM MgCl_2_, 0.5 mM Na_2_HPO_4_, 25 mM HEPES, 130 mM NaCl, 1 mM pyruvate, 5 mM d-glucose, and 0.1 mM CaCl_2_, pH 7.4 and the coverslips were mounted in a cuvette containing 3 ml of the nominally Ca^2+^-free assay solution (as above but 0.1 mM CaCl_2_ was replaced by 0.05 mM EGTA) and placed (at RT) in a spectrofluorimeter (Shimadzu, RF5001PC). The cells were treated with nucleotides (Sigma) applied at a concentration indicated in the relevant figures and 10 μM AR-C 118925XX (216657-60-2, TOCRIS Bioscience) applied 10 min before the addition of agonists. Fluorescence was recorded at 510 nm, with excitation at 340 and 380 nm. At the end of each experiment, the Fura 2 fluorescence was calibrated by the addition of 33 μM ionomycin to determine maximal fluorescence, followed by the addition of EGTA to complete the removal of Ca^2+^. Cytosolic Ca^2+^ concentration [Ca^2+^]c was calculated according to Grynkiewicz et al.^38^.

### Immunocytofluorescence (ICC)

Myoblasts were cultured on coverslips in the growth medium until 50–60% confluent. After rinsing twice with cold PBS (w/o calcium and magnesium), cells were fixed in a 4% w/v paraformaldehyde solution (PFA) in PBS for 15 min on ice and then permeabilized using PBS with 0.1% Triton X-100 for 5 min, blocked in 5% v/v goat serum (GS, Normal Goat Serum, S-1000, Vector Laboratories IVD) in PBS for 1 h at RT and incubated overnight at 4 °C with primary antibodies (aniP2RY1, antiP2RY2, anti-P2RY4 and antiP2RY6, as described above, diluted 1:100 in the blocking buffer). Myogenic cells were identified with the MYOD monoclonal antibody MA5-12902 INVITROGEN) diluted 1:100 in the blocking buffer, overnight at 4 °C. The secondary antibody (Alexa Fluor^®^488 goat anti-Rabbit, Thermo Fisher Scientific), diluted 1:1000 in 5% GS in PBS was incubated for 1 h at RT in the dark. Coverslips were rinsed 3 times for 10 min with agitation between each step of the ICC protocol. After staining, cells on coverslips were mounted onto microscope slides, sealed in Glycergel Mounting Medium with DAPI (H-1200 VectaShield^®^, Vector Laboratories) before imaging using a confocal microscope (Zeiss Spinning Disk Confocal Microscope) under conditions identical for both genotypes, and the image analysis was performed using ImageJ software.

### Random motility assay

15,000 myoblasts (*mdx* or w/t) were seeded into a 24-well cell culture plate and grown for 48 h in the culture medium. Time-lapse movies were generated by multiple well areas per each cell type being photographed every 15 min for 5 h 45 min using the inverted DMI6000 microscope (Leica Microsystems GmbH) equipped with an environmental chamber (PeCon GmbH) and a DFC350FXR2 CCD camera (Leica Microsystems GmbH). Both the bright field and the DIC Nomarski contrast using HC PL APO 10×/0.40 dry objective (Leica Microsystems GmbH) were captured. Acquired time-lapse movies were exported to TIFF format and aligned to compensate for possible drifts using an ImageJ plugin. Subsequently, at least 30 cells per each experimental condition were tracked semi-automatically in the time-lapse movies using the Track Objects plug-in in Leica MM AF powered by MetaMorph^®^ software (Leica Microsystems GmbH). Cells dividing or colliding with other cells were excluded from the analysis. The statistical significance was evaluated as described below.

### Data analysis

Data are expressed as a mean value ± standard deviation (SD). Statistical significance of pairwise comparisons between particular parameters in mdx myoblasts and their appropriate w/t equivalents was assessed by Student's t-test. A p-value of < 0.05 was considered statistically significant where n = 3–4 for PCR, n = 3–6 for Western blot data, and n = 3–5 for calcium measurements; “n” represents the number of repeated experiments with cells derived from three different mice.

### Ethics approval and consent to participate

All animals used in the experiments were kept at 20–24 °C, humidity 45–65% and a 12-h light–dark cycle. All efforts were taken to minimize the number of animals used and the amount of stress placed upon them. Experimental procedures complied with the Polish Law on Experimentation on Animals that implements the European Council Directive of 22 September 2010 (2010/63/UE) and the NIH Guide for the Care and Use of Laboratory Animals. The experiments were approved and controlled by the Animal Welfare Committee at the Nencki Institute of Experimental Biology, Warsaw, Poland.

We also confirm that the study is reported in accordance with ARRIVE guidelines (https://arriveguidelines.org).

## Results

### Transcripts analysis

**Figure 1:**
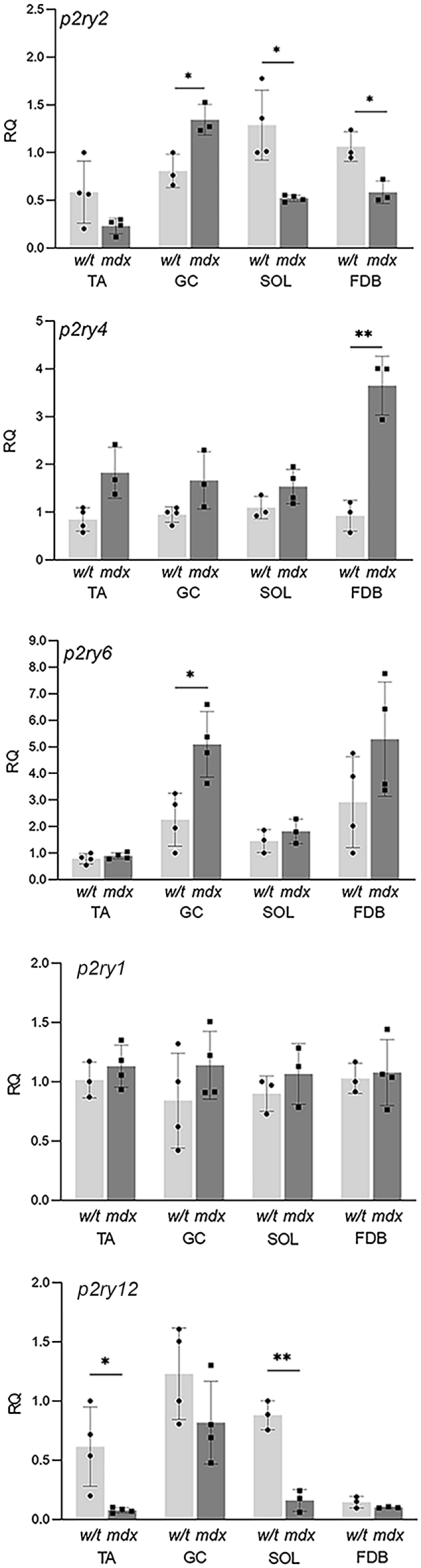
P2RYs transcript levels in myoblasts derived from Tibialis anterior (TA), Gastrocnemius (GC), Soleus (SOL) and Flexor Digitorum Brevis (FDB) muscles. Transcripts encoding P2RY2, P2RY4 and P2RY6 were tested in the same sample, thus their relative expression levels can be compared quantitatively., mRNAs encoding two ADP-activated receptors (P2RY1, P2RY12) were also detected in these samples. *p < 0.05, **p < 0.01 (mdx vs. w/t).

Expression analysis of specific p2rs (Fig. 1) showed that their mRNA levels differ between mdx myoblasts and their w/t equivalents isolated from the same muscle types. Moreover, the levels of specific p2r transcripts differ between different muscles from the same genotype, irrespective whether dystrophin-positive or dystrophic.

Furthermore, there was a great variability in the direction of these expression changes, with some receptors being significantly upregulated in one muscle and downregulated in another. Specifically, *p2ry4* and *p2ry6* were upregulated in dystrophic GC and FDB muscles, *p2yr2* expression was increased in GC but downregulated in FDB and SOL and *p2ry12* was consistently downregulated, but in TA and SOL only (Fig. 1). Of all the transcripts tested, the expression of *p2ry1* was the only one that remained unaltered across all muscles and both genotypes. Therefore, the effects of mdx mutation on the transcript levels of these metabotropic receptors do not allow for any generalisation. Analysis of individual qPCR traces indicated that expressions of the ADP-sensitive receptors were much lower than of *p2ry2* and *p2yr4*, while the transcript encoding P2RY13 was very poorly detectable and therefore not included in quantitative analyses (Supplementary 2).

### Protein expression analysis

**Figure 2:**
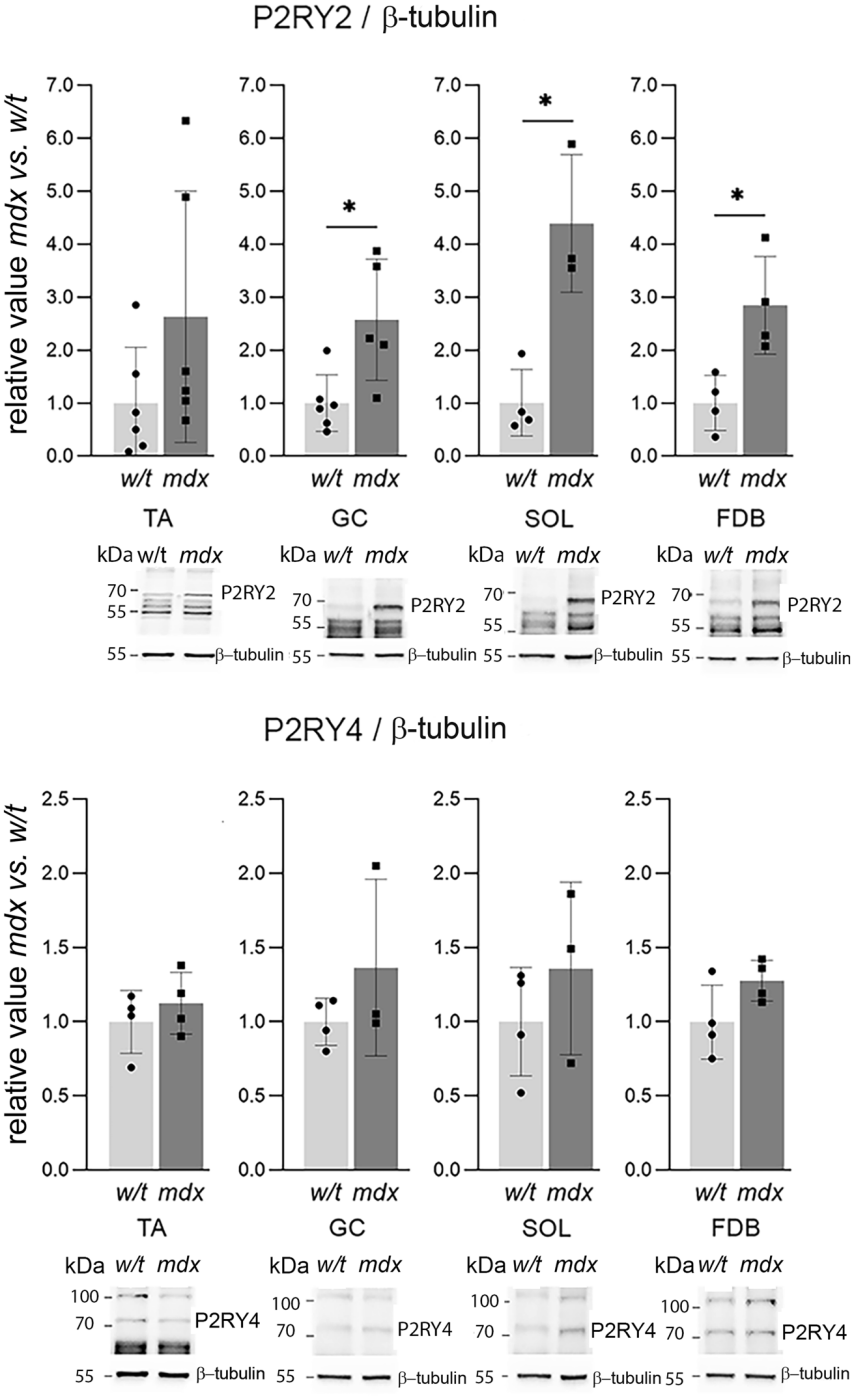
Western bloting alaysis of ATP-activated metabotropic receptors in primary myoblasts from TA, SOL, GC, FDB. Each bar represents the mean value from 3 independent experiments ± SD. Representative western blots are also shown. *p < 0.05 (mdx vs. w/t).

**Figure 3:**
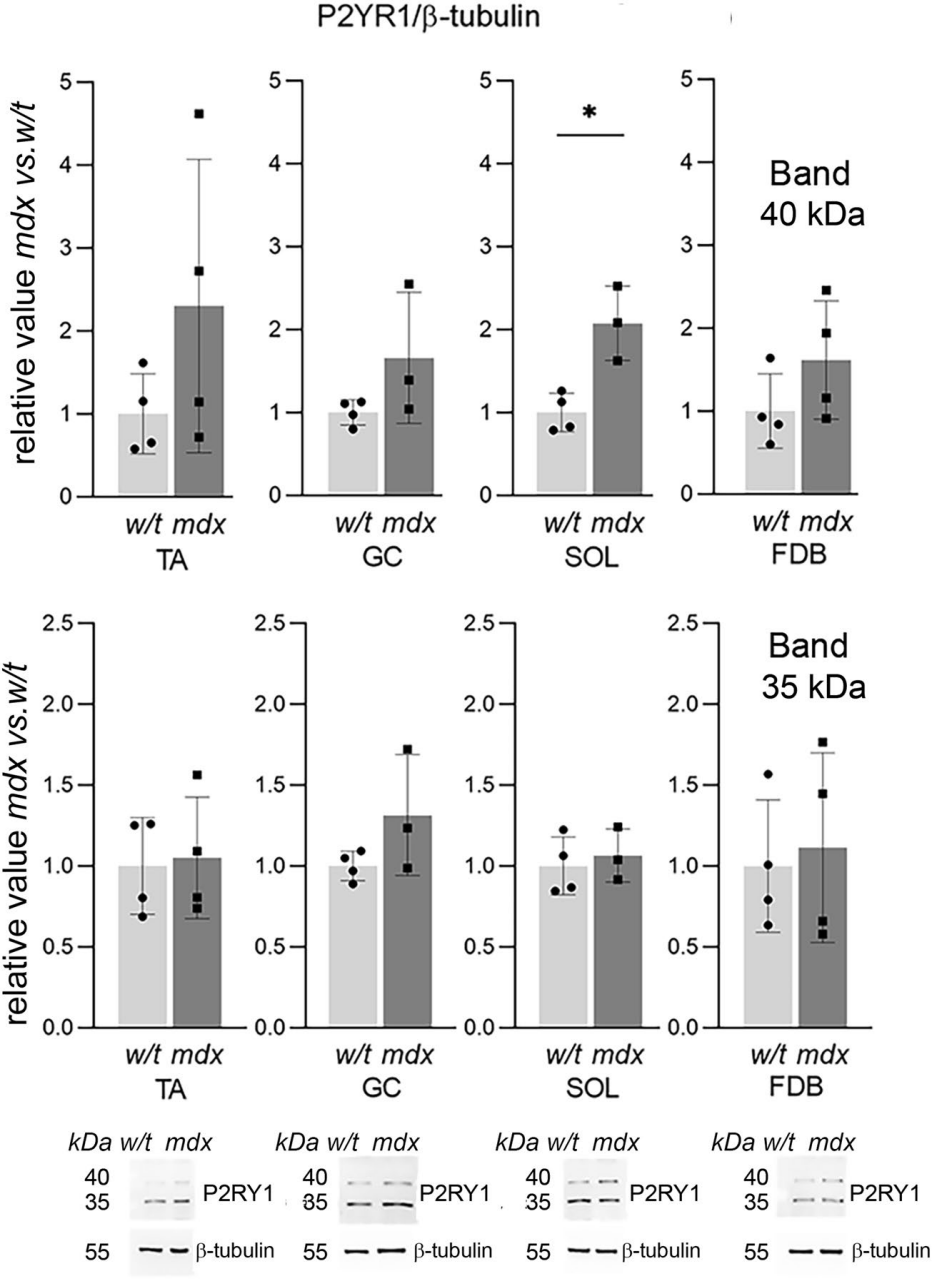
Western bloting analysis of ADP-activated metabotropic receptor P2RY1 in primary myoblasts. Each plot shows Western blot data quantification for the 40 kDa band (top) and 35 kDa band (bottom) Each bar represents the mean value from 3 independent experiments ± SD. Representative western blots are also shown. *p < 0.05 (mdx vs. w/t.)

**Figure 4:**
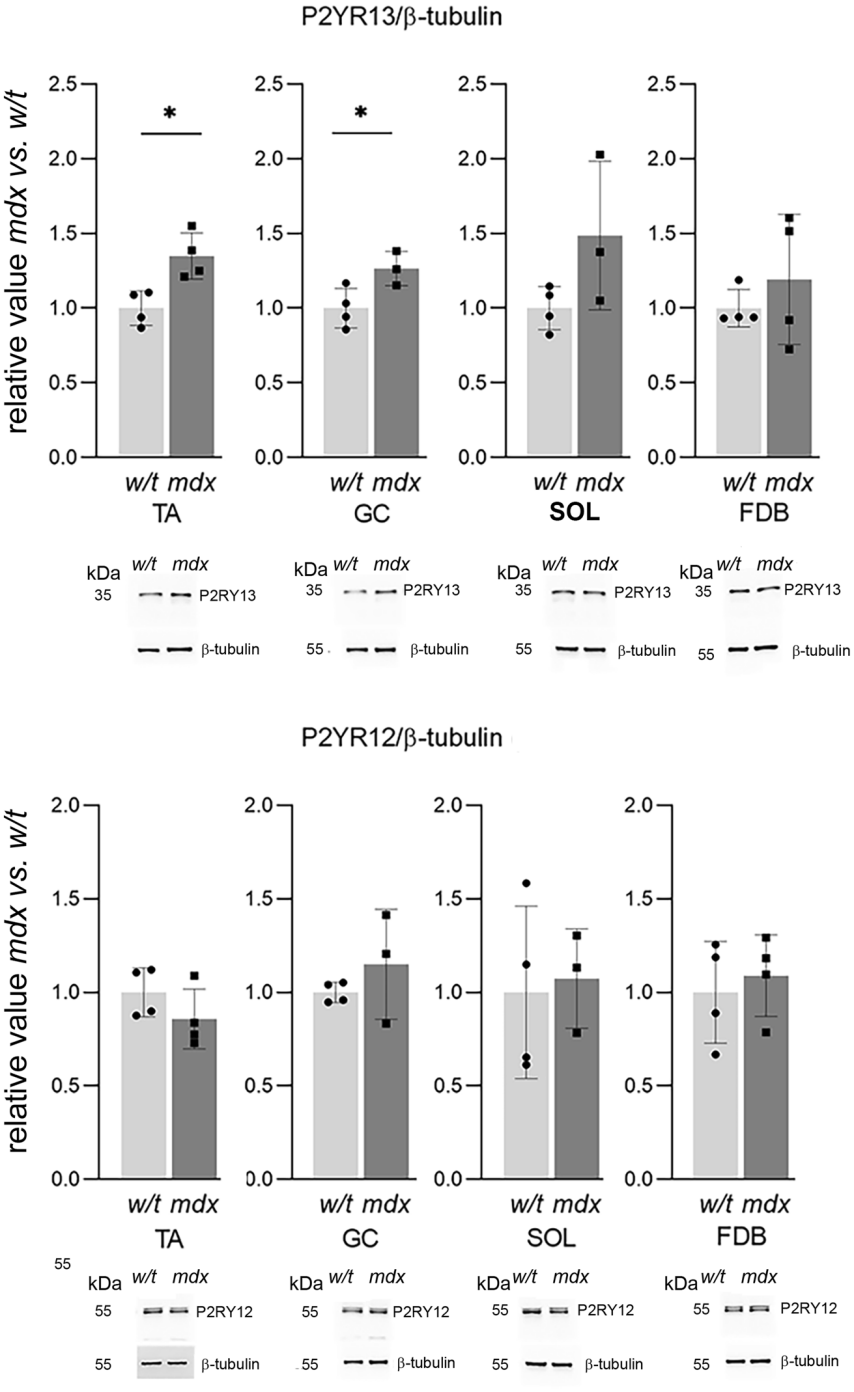
Western bloting analysis of ADP-activated metabotropic receptors P2RY12 and P2RY13. Each bar shows Western blot data from 3 independent experiments ± SD. Representative western blots are also shown. *p < 0.05 (mdx vs. w/t).

**Figure 5:**
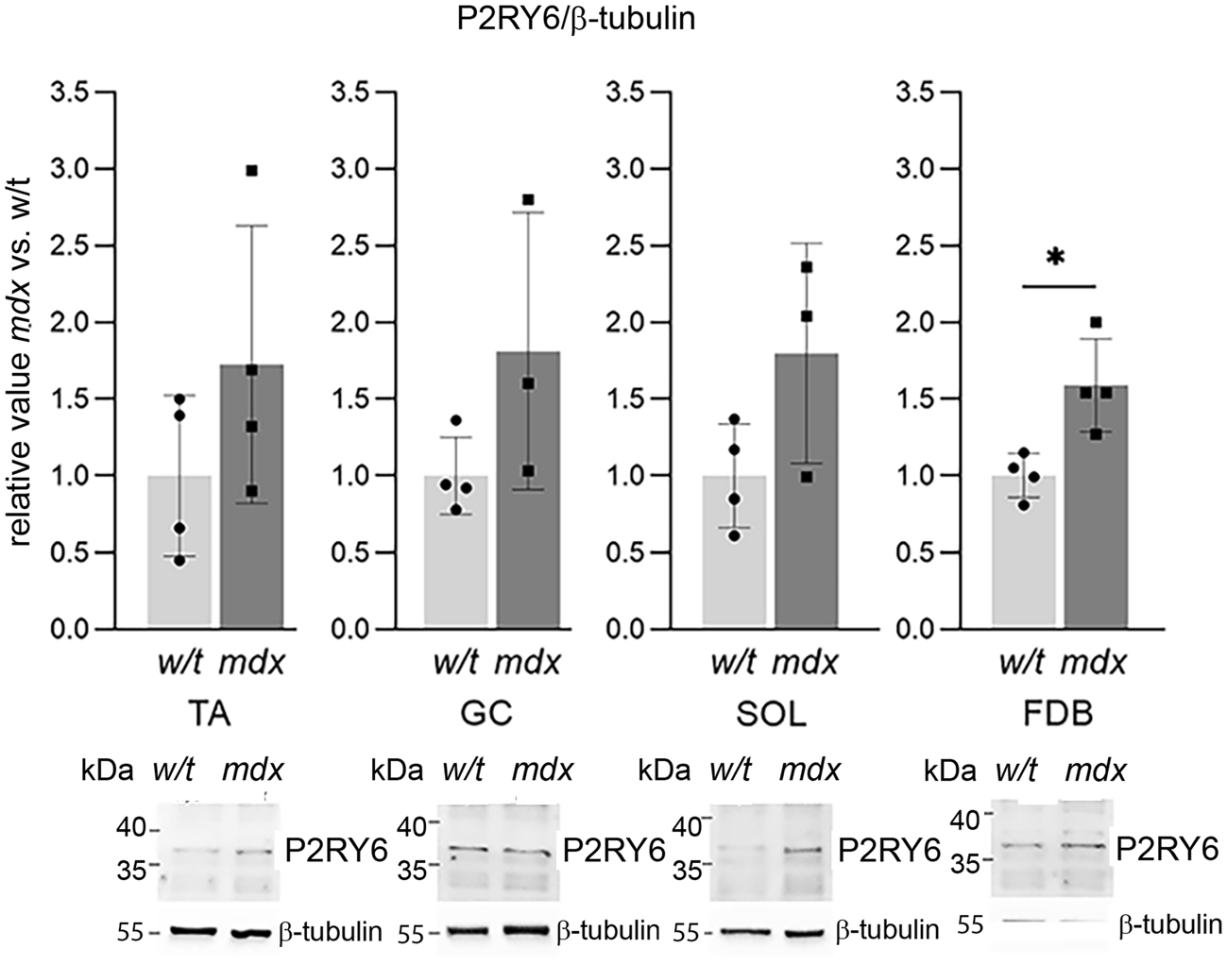
Western bloting analysis of UDP-activated metabotropic receptor P2RY6. Each bar represents the mean value from 3 independent experiments ± SD. Representative western blots are also shown. *p < 0.05 (mdx vs. w/t).

In contrast to the mRNA expression variability, alterations at the protein level were consistent for all 6 receptors, and either elevated or found unchanged in mdx myoblasts isolated from all muscles tested (Figs. 2, 3, 4, 5). Particularly, the level of P2RY2 protein was substantially increased in myoblasts, which could suggest a specific impact of P2RY2 on calcium signalling in these cells (Fig. 2). Noteworthy, mdx mutation never resulted in the reduced protein levels in myoblasts isolated from any of these muscles.

**Figure 6:**
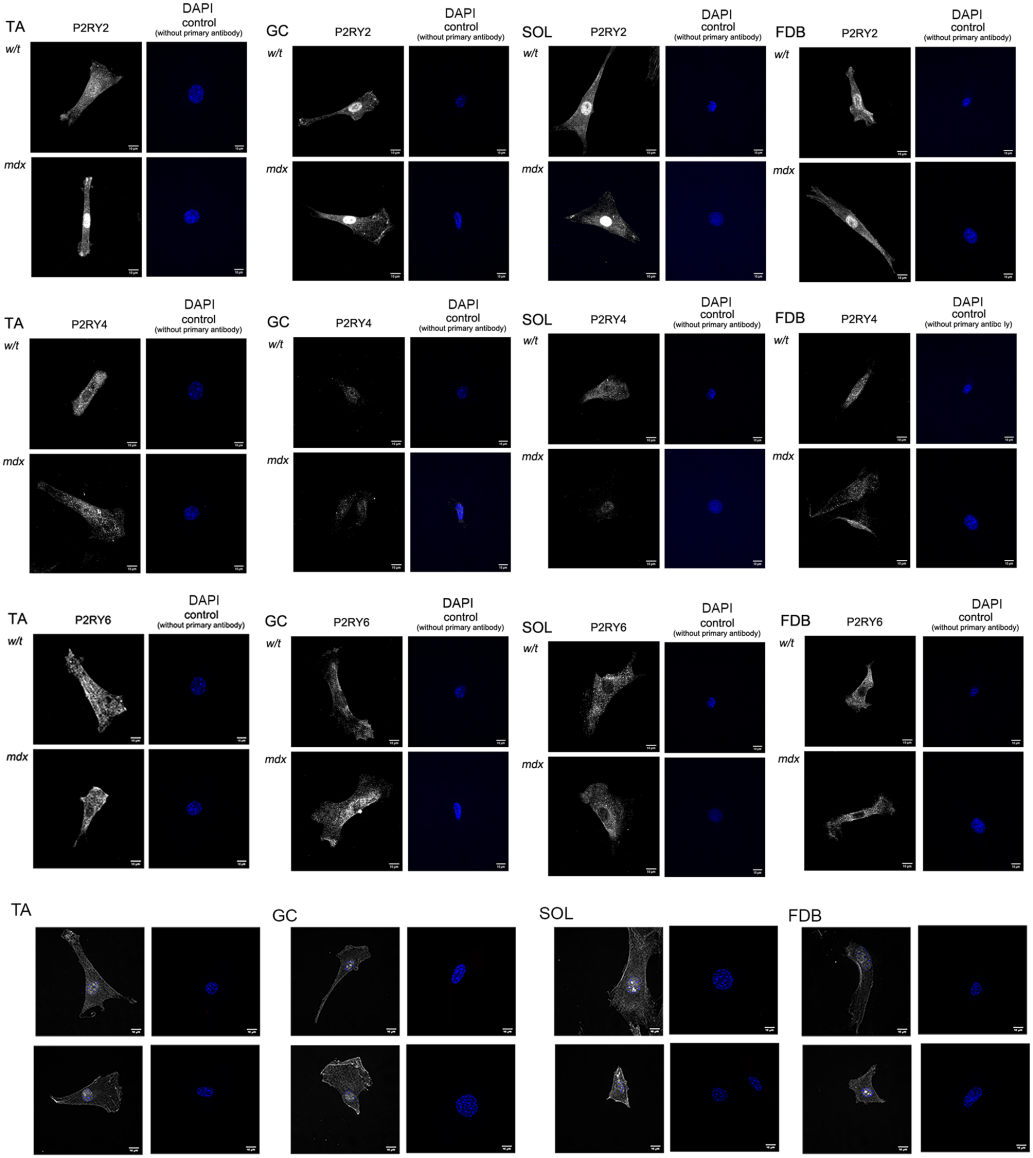
Fluorescent microscopy visualization of P2RY2, P2RY4, P2RY6 and P2RY1 in myoblasts isolated from TA, GC, SOL and FDB muscles of w/t and mdx mice. Representative example pictures of cells stained with the specific antibody, and secondary antibody only negative controls counterstained with DAPI are shown. Size bar = 10 µM.

Further clarification of the potential involvement of these metabotropic receptors was sought following their cellular localisation using immunofluorescent detection (Fig. 6). While the localisation of P2RY2 was consistent with its presence in the plasma membrane, the quantitative differences between w/t and mdx cells in SOL were not strong enough to be considered as corresponding with the Western blotting data, showing higher levels of this receptor in SOL extracts. Interestingly, P2RY4 seems to localise inside the cells, regardless of their muscle origin (Fig. 6).

In addition, in mdx myoblasts obtained from TA and GC, more P2RY2 seems to localise to the plasma membrane than in w/t cells. In SOL and FDB, no differences in membrane localisation could be clearly determined. The nuclear staining with the P2RY2 antibody correlates with its sub-cellular localisation observed previously in immortalized myoblasts^32^. It seems to reflect a nonspecific signal produced by this antibody, as we have previously shown by Western blotting of proteins extracted from isolated nuclei. When probed with P2RY2 antibodies, a smaller molecular weight band was detected, which was insensitive to P2RY2 gene silencing^32^. P2RY1 and P2RY6 also differ in their intracellular localisation in myoblasts from different muscles, but the effect of mdx mutation on their intracellular distribution was not obvious. Two bands of P2RY1 presumably represent two isoforms of this receptor or posttranslational modifications. Similar results were also shown by others^39^.

### Calcium release from the ER stores

**Figure 7:**
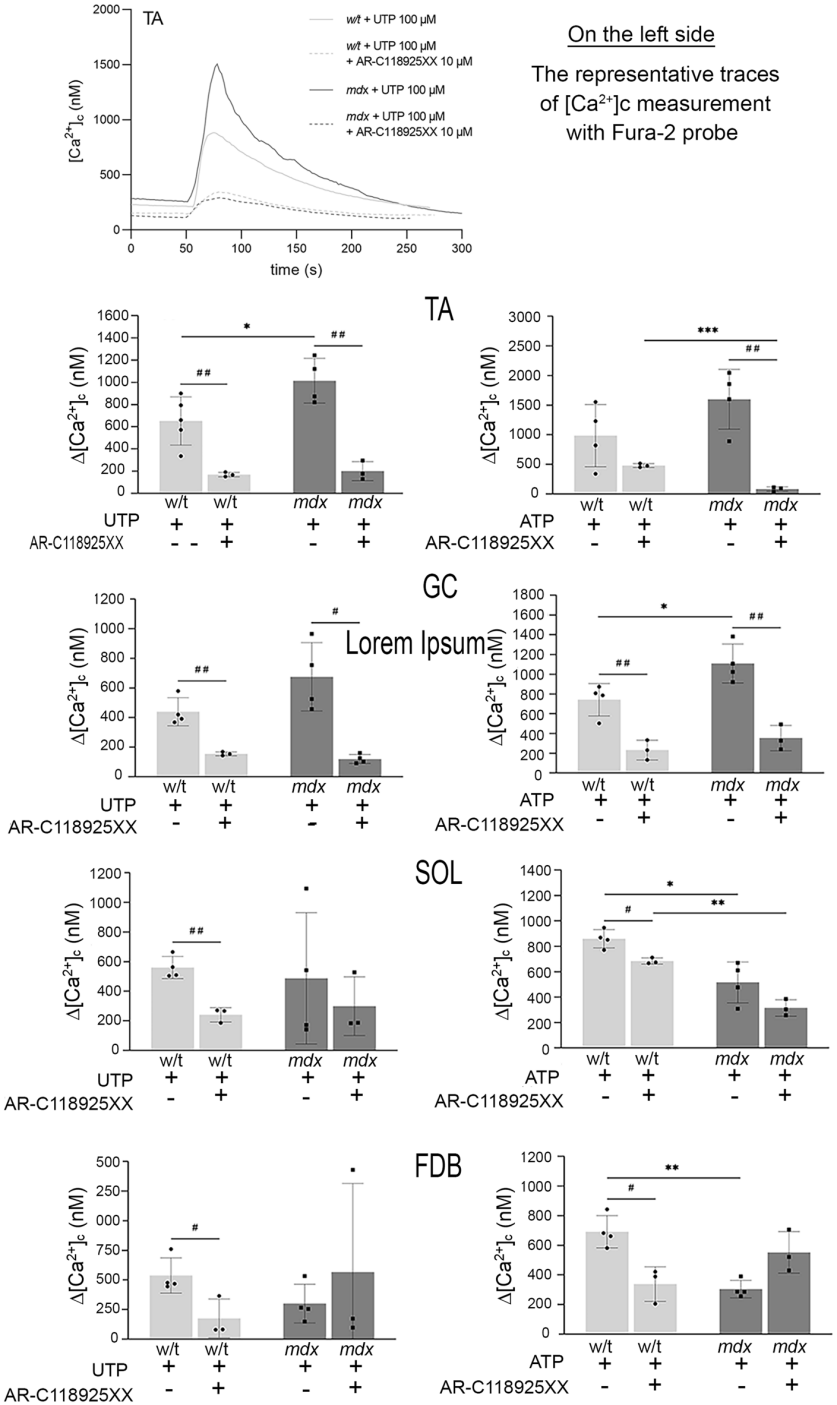
Nucleotide-induced calcium release from ER. The representative trace (Top) is shown to illustrate the general concept of the experiment. Myoblasts were incubated with or without P2RY2 inhibitor (AR-C118925XX) and then 100 µM UTP or 500 µM ATP was added. Data collected from three independent experiments are shown. Asterisk (*) marks statistically significant differences: mdx vs. w/t and (mdx + P2RY2 antagonist) vs. (w/t + P2RY2 antagonist). Hashtag (#) marks differences: mdx vs. (mdx + P2RY2 antagonist) and w/t vs. (w/t + P2RY2 antagonist). * and # p < 0.05; ** and ## p < 0.01; *** p < 0.001.

**Figure 8:**
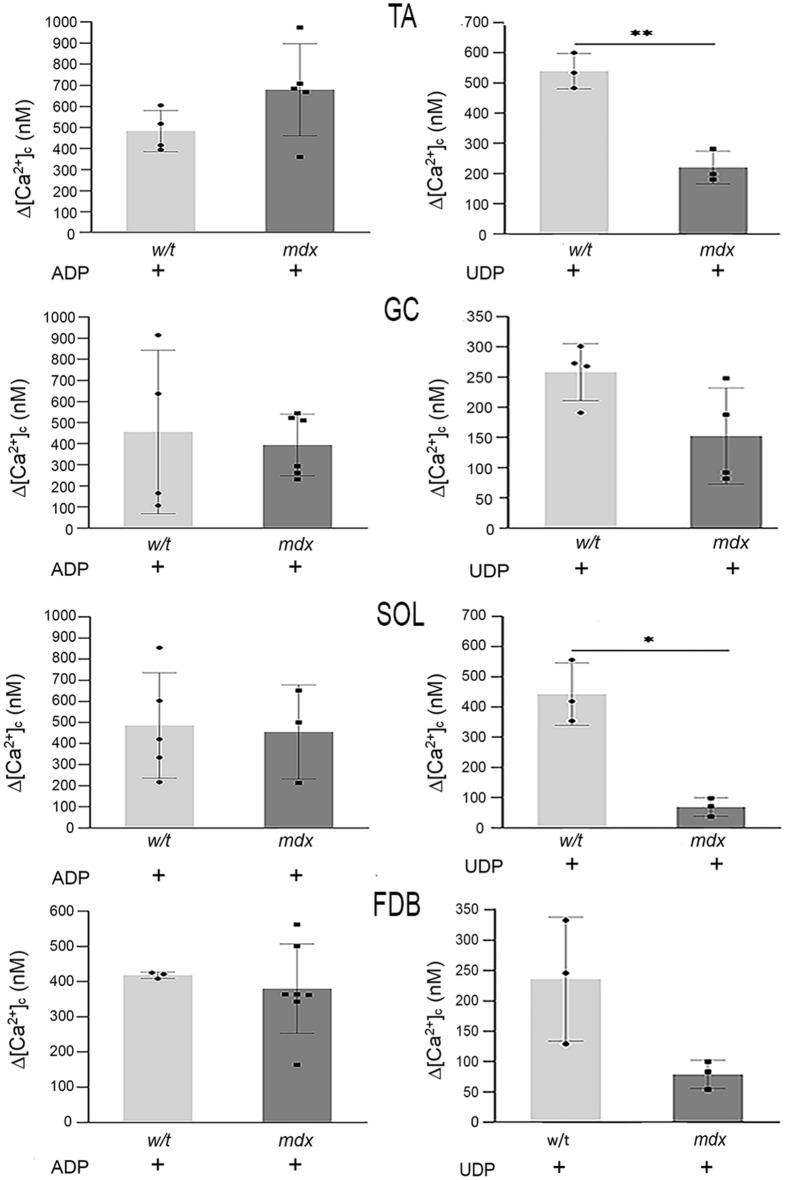
Nucleotide-induced calcium release from ER. The protocol is the same as shown in Fig. 7. Myoblasts were stimulated with 1 mM ADP or 1 mM UDP. Data collected from three independent experiments are shown. Asterix(s) * marks mdx vs. w/t. *p < 0.05; **p < 0.01.

Stimulation of metabotropic purinoceptors results in Ca^2+^ release from the ER-calcium stores. In the presence of external calcium, this step is followed by Ca^2+^ influx into cells. In the absence of extracellular calcium, a transient increase in the cytosolic Ca^2+^ concentration results solely from the depletion of calcium stores and is the only measure of the cellular calcium response. Under such conditions, the calcium response is unaffected by further signalling steps, such as the store-operated calcium entry, secondarily leading to the elevation of cytosolic Ca^2+^ concentrations. Therefore, we tested the effects of specific purinergic agonists under the Ca^2+^-free conditions (Figs. 7, 8).

We found that, in w/t myoblasts, the intensity of calcium response to particular agonists, visualised as a height of cytosolic [Ca^2+^] peak immediately after stimulation, is similar regardless of their muscle origin. In contrast, the intensity and direction of dystrophy-induced changes in cytosolic Ca^2+^ concentrations upon stimulation with the same agonist could be different in myoblasts from various muscles and do not always reflect changes in the corresponding transcript and protein levels. The most unequivocal dystrophic alteration of Ca^2+^ response was observed in myoblasts derived from TA and GC treated with ATP or UTP (Fig. 7). In contrast to this, the mdx mutation weakened the response to the same agonists in myoblasts isolated from SOL or FDB muscles. The residual calcium response to ATP or UTP stimulation observed in cells preincubated with P2RY2 antagonist may reflect the P2RY4 activity. The reduced calcium response to UTP and ATP in mdx cells derived from FDB is difficult to explain, as the levels of corresponding transcript and protein were found to be substantially elevated. It is noteworthy that the activity of UDP-responsive P2RY6 was significantly reduced in all mdx myoblasts tested, while this mutation did not affect ADP-evoked responses in myoblasts from any of the four muscles tested (Fig. 8). ADP is a common agonist for P2RY1, P2RY12, and P2RY13. Because we have not analysed the effects of specific antagonists, we cannot discriminate between them.

### Cell motility

Previous studies in immortalized, as well as primary dystrophic myoblasts, showed that dystrophinopathy can affect cell motility^9,32^. Given that this function is important for myoblasts to be able to reach the site of muscle damage, and that intracellular calcium changes affect cell movement, we investigated the potential role of metabotropic receptor alterations in this process.

**Figure 9:**
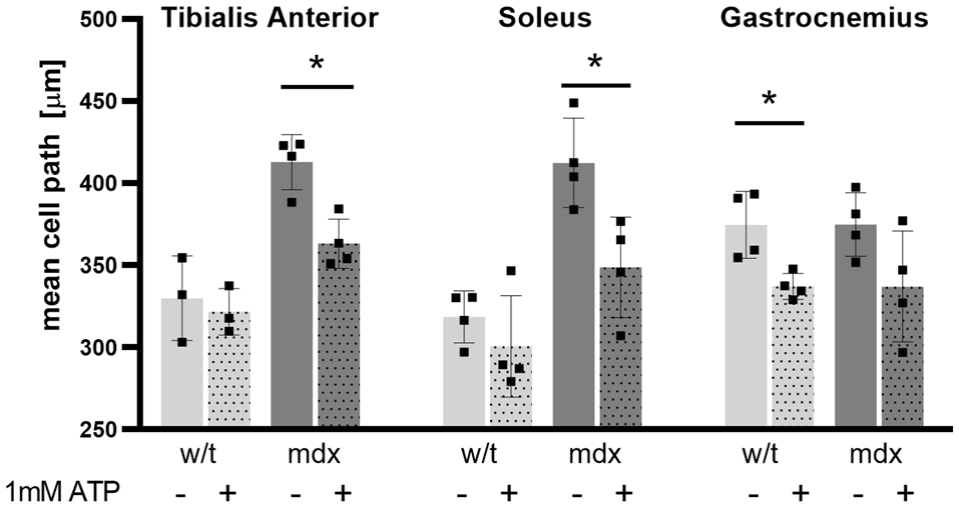
Random motility of primary myoblasts; effects of ATP-evoked cell stimulation. Data collected from cells isolated from 4 mice, with 30 myoblasts per muscle type being tracked. Differences in distance covered (µm) are shown *p < 0.05.

As shown in Fig. 9, the random motility of unstimulated primary myoblasts isolated from dystrophic TA and SOL was substantially increased in comparison to w/t cells. This observation is in line with previously published data for the dystrophic myoblast cell line^32^. Contrary to cells from TA and SOL, the motility of w/t and mdx myoblasts isolated from GC was similar. Also, the motility of TA- and SOL-derived dystrophic myoblasts was substantially reduced upon treatment with ATP but only slightly affected in GC myoblasts. In contrast, ATP only slightly reduces the motility of TA- and SOL-derived w/t myoblasts while is more efficient in w/t GC-derived cells. The mechanism of myoblasts excitation with ATP cannot be attributed to specific receptors, as this agonist not only activates metabotropic P2RY2 and P2RY4 but also acts on the ionotropic P2RX7, which effect was documented earlier. Nevertheless, these results demonstrate that the muscle-specific imprinting of myoblasts impacts yet another important cell function found to be altered by the absence of dystrophin.

### Cellular calcium toolkit

**Figure 10:**
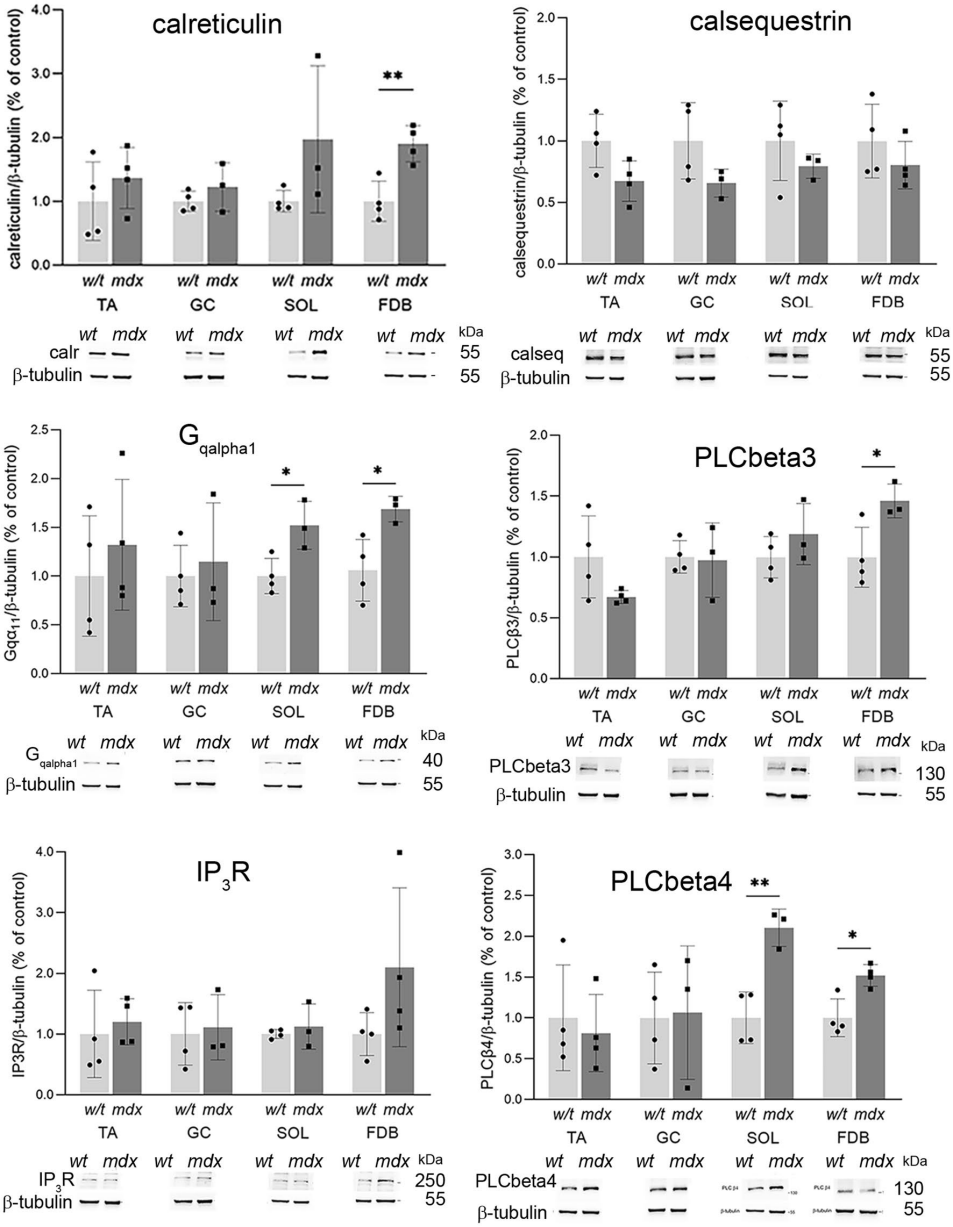
Western blot analysis of selected proteins belonging to the calcium toolkit involved in the generation of calcium signal analysed in myoblasts isolated from TA, GC, SOL and FDB. Bars represent mean values for three independent experiments ± SD. Below each graph data from one representative western blot experiment are shown. *p < 0.05, **p < 0.01.

**Figure 11:**
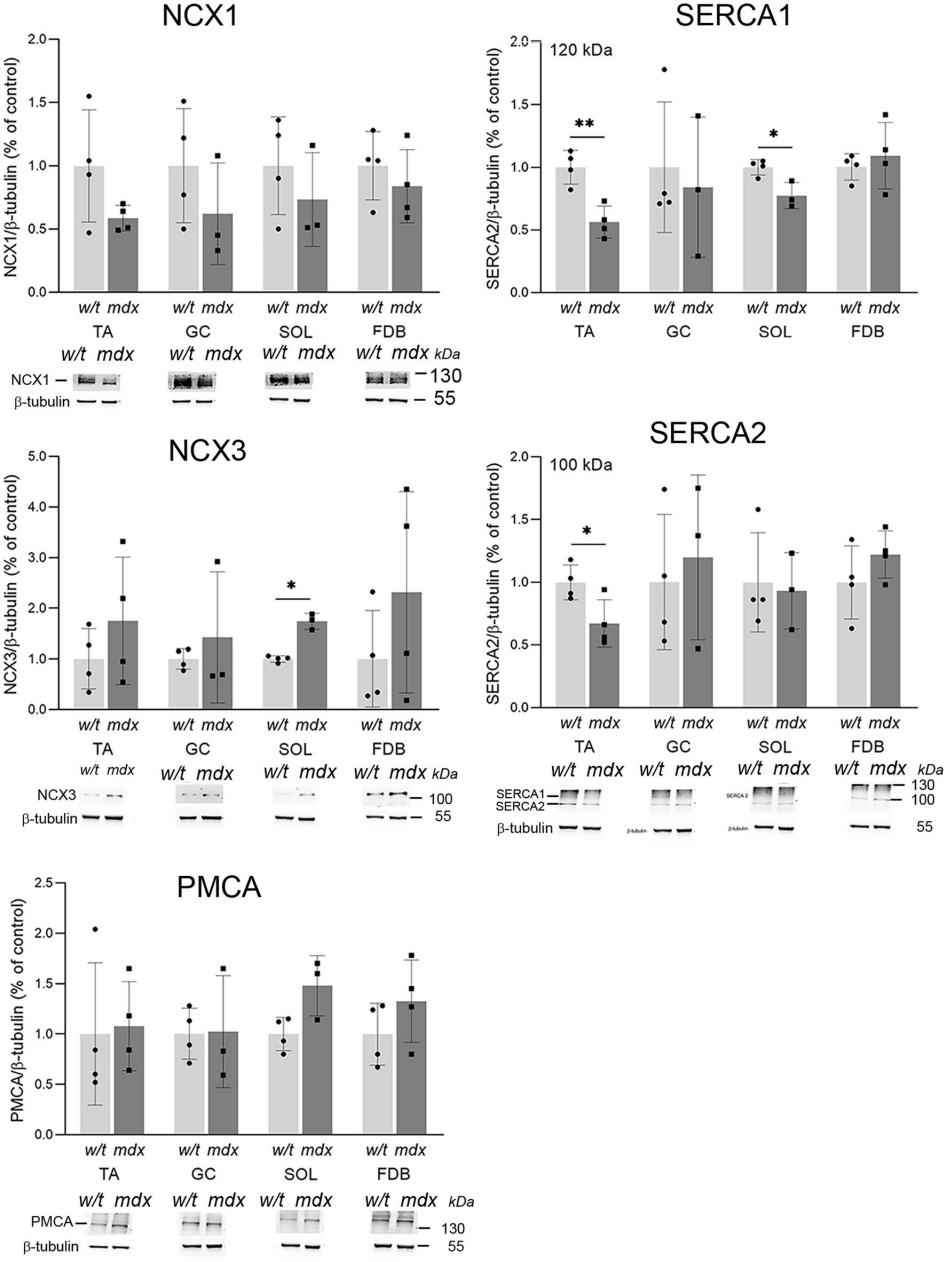
Western blot analysis of selected proteins belonging to the calcium toolkit involved in the reduction of calcium signal analysed in myoblasts isolated from TA, GC, SOL and FDB. Mean values from three experiments ± SD are shown. Representative western blots are shown. *p < 0.05, **p < 0.01.

We have shown previously that the increased susceptibility of the immortalised mdx myoblasts to metabotropic purinergic stimulation is not the only explanation for elevated Ca^2+^ concentration in these cells upon treatment with specific nucleotides. The final shape of the cellular response (its spatiotemporal profile), which is crucial for the proper cellular decoding of calcium signals, is modulated by a broad spectrum of “calcium toolkit” proteins, their levels, activities and interactions. These factors should be taken into consideration when the physiological consequences of elevated sensitivity of cells to extracellular nucleotides are considered. Proteins belonging to the calcium toolkit may be attributed to two main categories. These are proteins involved in the generation and maintenance of the Ca^2+^ signal (Fig. 10) and proteins that terminate cellular calcium responses (Fig. 11).

In primary myoblasts, we discovered that the effects of mdx mutation were, with rare exceptions, indistinguishable in all muscles tested. The notable exceptions were the upregulations of calreticulin, Gqα1 and PLCβ 3 and 4 in FDB (Fig. 10]. These proteins are involved in the generation of cellular calcium response upon stimulation by extracellular factors, thus this observation is in line with the general concept of elevated Ca^2+^ concentration and intensified calcium responses in dystrophic muscle cells.

## Discussion

Altered calcium homeostasis is a common feature in many different cells harbouring mutations in the DMD gene, regardless of whether dystrophin protein is expressed at detectable levels in their respective wild-type counterparts^16^ Previous studies revealed that myoblasts with the mdx mutation are more susceptible to ATP stimulation, mainly because of the increased activity of P2RY2 and P2RX7 purinoceptors. Both metabotropic and ionotropic ATP-sensitive nucleotide receptors produce a similar effect, which is an elevation of cytosolic Ca^2+^ concentrations. While the significance of the P2RX7 upregulation for the dystrophic pathology has been confirmed by the therapeutic impact of its genetic ablation and pharmacological inhibition^28,29,40^ the involvement of metabotropic purinoceptors is not that well understood. Yet, it might be important both for understanding the pathology and also as a potential therapeutic target. Indeed, the exacerbated ATP-evoked calcium response in dystrophic myoblasts results from substantial alterations of a broad spectrum of “calcium toolkit” elements, including calcium pumps, exchangers and buffers^16,32^. Potentially, some of these receptors and regulatory proteins could be good therapeutic targets in this debilitating disease. Notably, many alterations occur not in myofibers, currently considered to be the main muscle cells affected by DMD, but in myoblasts. Recent molecular and functional alterations found in dystrophic myoblasts confirmed that myogenic cells are affected by DMD^9^.

Many of the purinergic alterations were studied in established dystrophic myoblast cell lines maintained long-term in culture. The present study aimed at testing whether primary myoblasts isolated from individual muscles would present the same or rather a muscle-specific purinergic phenotype. We examined cells derived from TA, GC, SOL and FDB leg muscles. Irrespective of whether each of the P2Y receptors is considered separately across all muscles or all receptors in one muscle are compared, the pattern of expression and functional significance of P2RYs amongst w/t myoblasts derived from these four muscles differ. Moreover, the effects of mdx mutation on P2RYs expression and activity are also diverse across these muscles. Finally, functional responses are not always corresponding with changes in receptor protein levels. It is likely that similar or even greater differences exist between other muscle groups throughout the body. Interestingly, this finding helps our understanding of the potential mechanism of purinoceptor upregulation in dystrophic cells. Although it was tempting to associate it with the loss of dystrophin anchoring, resembling the DAP complex dys-regulation, this mechanism is unlikely. Firstly, there is no evidence of a direct or indirect interaction between dystrophin and any of the several purinoceptors involved. Secondly, these alterations also occur in myoblasts and lymphoblasts^10^ cells that do not express detectable dystrophin protein, and abnormalities found in myoblasts are likely to be epigenetic^9^. Finally, the muscle-specific variability described by us here cannot be reconciled with the absence of dystrophin across all these muscles. Therefore, the most likely explanation for this dystrophic purinergic phenotype is a compensatory adaptation to unfavourable conditions: reactive oxygen species, inflammation and metabolic abnormalities, which are present in dystrophic muscles. It resembles the overexpression of P2RX7 on different cancer cells, where stimulation of this receptor provides significant pro-survival benefits, including increased growth, migration and invasion, and the Warburg effect (reviewed ^41^). Indeed, the impacts of DMD on muscle cell energetics have been described^9,18,42^. It is unclear whether these mitochondrial alterations are a causative metabolic defect or adaptive reprogramming^43^. Purinergic modulation might be one of the mechanisms compensating for the altered cell metabolism, calcium homeostasis and energy demands. In addition to ATP receptors, altered ATP breakdown to adenosine by ecto-ATPases adds further complexity to the purinergic signalling cascade: Activation of adenosine-selective receptors is broadly immunosuppressive^44^, and also shown to protect skeletal muscle against injury^45^. However, the involvement of adenosine receptors in DMD has not been studied.

The origin of muscle-specific differences described here is unknown, but it may reflect unique metabolic features of particular muscles and also changes in the local dystrophic environment. As mentioned, isolated muscle cells appear to remember the in vivo environments from which they are derived^36^, and epigenetic factors seem to play a key role in this process^36^. While the limited spectrum of muscles tested here does not allow for any generalisation regarding slow-twitch vs. fast-twitch sensitivity to dystrophinopathy in relation to metabotropic receptors, this question seems worth addressing in the future.

Here we have extended previous data indicating faster random motility of mdx myoblasts than the w/t equivalents and the inhibitory effect nucleotides have on the migration velocity of mdx but not w/t cells. These effects were observed in TA and Soleus but not in GC muscle, further highlighting differences between myoblasts derived from different muscles of the same animal. The inhibitory effect of ATP on cell motility is not limited to dystrophic myoblasts. Kobayashi et al.^46^ found that extracellular ATP inhibits naive T cell migration in resting lymph nodes. Furthermore, ATP inhibits breast cancer^47^, and human endometrial cells migration^48^. Given that migration to the site of damage is an important factor in muscle regeneration, the significance of faster motility of mdx cells and the inhibitory effect of the purinergic agonists UTP^32^ and ATP on mdx motility should be considered in this context.

The biochemical explanation of the ATP effects on the migration of dystrophic myoblasts is not clear. This phenomenon can result from the substantial elevation of intracellular Ca^2+^ concentration in dystrophic myoblasts. However, such a putative link is not a common fact for all muscles tested. Furthermore, it must be noted that the intensities of the calcium response shown in Figs. 7 and 8 were estimated in the Ca^2+^-free solution, while motility was observed in the standard Ca^2+^-containing medium. Such a difference between the experimental protocols was unavoidable, but it makes data interpretation more difficult. Stimulation of cells with ATP in the presence of extracellular calcium may have at least two additional effects related to calcium response. Firstly, the depletion of the ER-calcium stores upon treatment of cells with ATP would activate store-operated calcium entry. Secondly, ATP activates P2RX7, which is a plasma membrane Ca^2+^ channel overexpressed in dystrophic myoblasts (17). Both events could dramatically affect Ca^2+^-dependent cellular behaviour, including cell motility. Thus, in this work we have characterized the very basic cellular calcium response that is receptor-activated Ca^2+^ release under Ca^2+^-free conditions. This phenomenon may be used as the simplest indicator of the individual activity of specific P2RY but not of the global cellular calcium response. Regarding the functional relevance of such a cellular response, it could slow down myoblast migration away from the areas of locally-increased eATP concentration to promote regeneration.

The most evident mdx-evoked change within the P2Y receptor family concerns the upregulation of the P2RY2, which is identifiable in myoblasts derived from all muscles tested. This is also the only P2RY whose activity is elevated (albeit variably) in mdx myoblasts regardless of their muscle of origin. Moreover, findings in primary myoblasts agree with those on P2RY2 in immortalized mdx myoblasts^32^. Thus, immortalized myoblast may be considered as a valuable model for basic biochemical studies to identify intrinsic properties distinguishing w/t and mdx cells. On the other hand, the pattern of expression of proteins belonging to the “calcium toolkit” differed between primary and immortalized myoblasts^32^. Interestingly, even in primary cells, some calcium toolkit proteins were found altered consistently across all experimental conditions and cells, while others showed greater variability between experiments (Figs. 10 and 11). Given that all proteins were analysed concomitantly in the same lysate, poor reproducibility in only some of them may suggest that their cellular levels did vary significantly. Indeed, the cellular level/activity of proteins that have crucial regulatory functions must be controlled more precisely than proteins which are less critical for the global regulation of the cellular metabolism, therefore their levels may be controlled less tightly. This effect would be greatly influenced by adaptations to long-term culture conditions, as it is seen in cell lines.

In this context, it is worth noting that the immortalized muscle cells, such as the commonly used C2C12, have an undefined muscular origin and/or potentially suffer from the “loss of the memory of origin” concerning the properties of the particular muscle that they were isolated from. On the other hand, the very procedure of primary myoblast isolation, which is inseparable from oxidative stress, may also have an uncontrollable impact on these cells. Therefore, experimental approaches using both cell lines and primary cells are useful and complementary, whilst none of them alone is free from limitations. Moreover, any effects observed in immortalized cells or primary myoblasts isolated from a specific dystrophic or w/t muscle should not be unquestionably generalized and translated directly to myoblasts across all muscles. Moreover, the homogeneity of primary cells in culture is more difficult to be maintained as they exhibit a strong tendency to enter the differentiation process, which kinetics differs between dystrophic and w/t cells^8^. This may explain a lower level of reproducibility in some studies.

## Conclusions

Analyses in primary myoblasts isolated from various leg muscles confirmed abnormalities of metabotropic P2Y2 receptor expression and function and of calcium signalling, previously identified in immortalized dystrophic myoblasts. These data demonstrate that the purinergic abnormality in undifferentiated muscle cells is a true consequence of dystrophinopathy, which persists in cultured cells, despite all the adaptations to in vitro conditions.

Importantly, while myoblasts derived from distinct muscles exhibit the key underlying pathological abnormalities involving calcium signalling, the pattern of changes across cells isolated from various muscles is similar but not identical. It is important, as experiments in mouse and human myoblasts isolated from one muscle type may not be representative for all dystrophic cells. Moreover, these differences may impact therapeutic myoblast transplantation. Our results broaden our understanding of both DMD pathology and muscle cell biology.

## Supplementary Information

Supplementary Information.

## Data Availability

The preliminary datasets generated in the current study are available as a preprint in BioRxiv. Raw data are available from the corresponding author on reasonable request.
