# Supplementary material for: Primary mouse myoblast metabotropic purinoceptor profiles and calcium signalling differ with their muscle origin and are altered in mdx dystrophinopathy
Source: Sci Rep. 2023 Jun 8;13:9333. doi: 10.1038/s41598-023-36545-y (PMC10250391; doi:10.1038/s41598-023-36545-y)
Supplement: Supplementary file 1 — Supplementary Information. [file 41598_2023_36545_MOESM1_ESM.pdf]

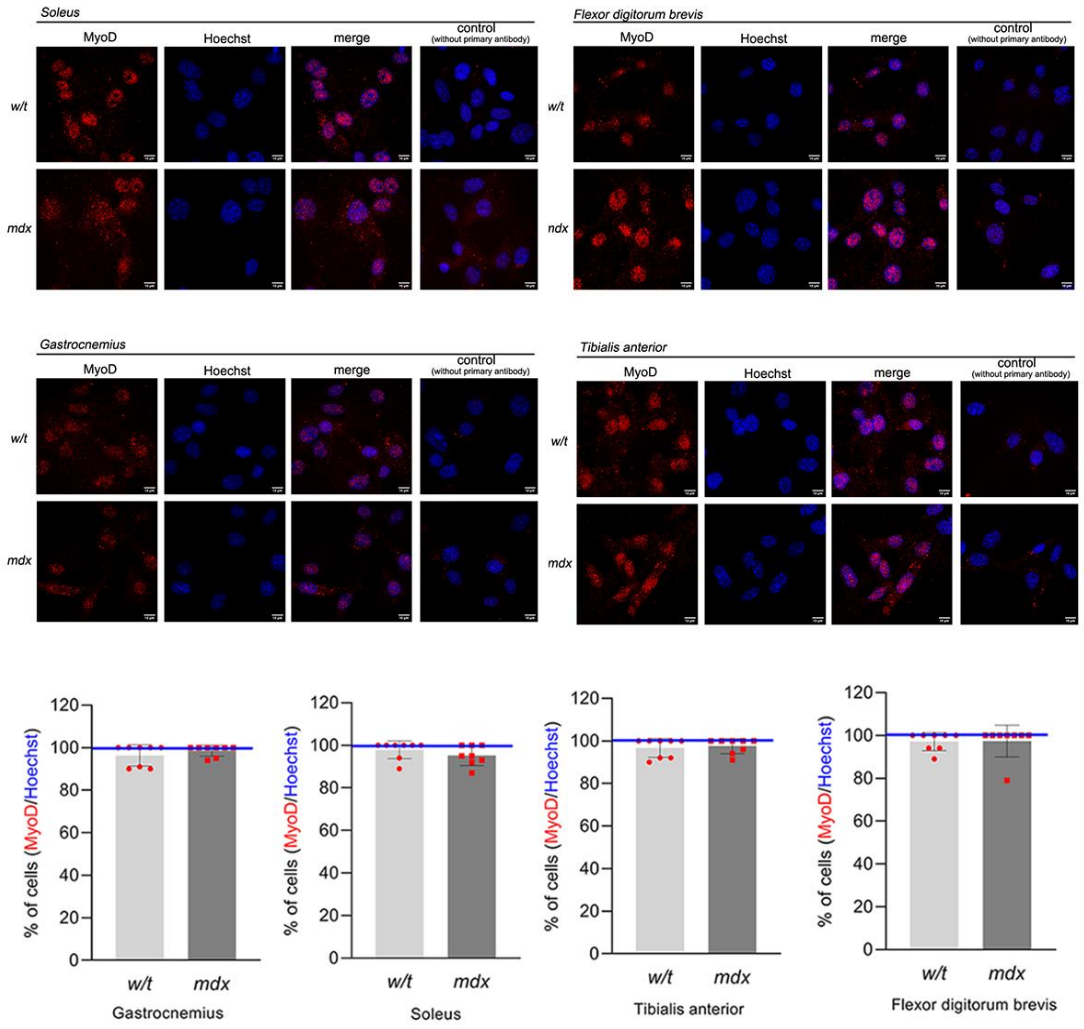

Suppl. 1 The purity of w/t and mdx myoblast cultures isolated from TA, GC, SOL and FDB muscles.

Upper panels: MyoD staining (red signal – positive cells) and Hoechst labelling (blue signal - nuclei) in primary myoblasts. Representative immunofluorescent image from eight biological replicates is shown.

Lower panel: The charts show the percentage of MyoD-positive cells (red signal) in comparison to the total number of cells based on nuclei labelling (Hoechst,blue signal). Analysis was performed using ImageJ software. The purity of w/t and mdx myoblast cultures was consistently > 94 %. Error bars represent the mean number of cells with SD deviation for eight images. There was no statistically significant difference in the MyD positive cells between w/t and mdx cultures (Student T-test)

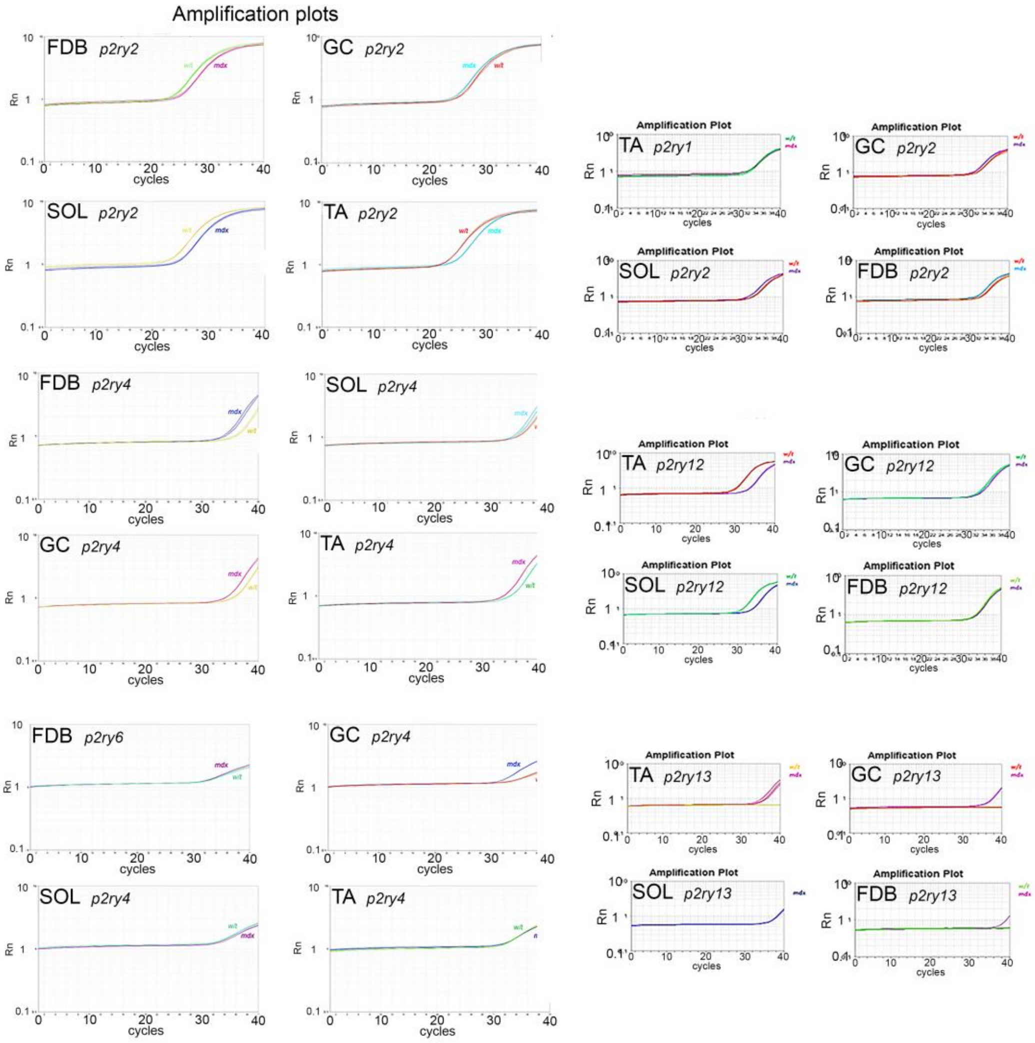

Suppl. 2 qPCR amplification plots showing the number of cycles necessary to detect transcripts of genes encoding metabotropic nucleotide receptors in myoblasts isolated from TA, GC, SOL and FDB.

Suppl. 3

Western blot analysis of P2Y receptors and loading controls.

P2Y receptor analyses in primary myoblasts from w/t and mdx animals were performed in cells isolated from 3 individual animals. Duplicate samples containing the same amount of protein were resolved in two gels, transferred and probed simultaneously. This approach was taken because the sizes of some P2Y receptors of interest and proteins used as loading controls (because of their stable expression across cells from the two genotypes and different muscles) overlap in range, and that blot stripping introduces significant errors. Therefore, specific receptors were detected in one blot and β-tubulin, used as the loading control, was detected in the duplicate blot. Both procedures were done simultaneously.

Western blots of β-tubulin used for analyses of **P2RY1, P2RY12 and P2RY13.**

Blots show β-tubulin detected in lysates obtained from myoblasts isolated from TA, GC, SOL and FDB muscles of three w/t and mdx mice. Each lane represents lysate prepared from myoblasts from a specific muscle isolated from one animal. Protein mass standards are also shown as the white ladders.

These blots were exposed to the intensity just before saturation, so the band could be used to confirm the equal protein loading in each well and therefore allow for the estimation of the relative amounts of P2RY1, 12 and 13 receptors.

The identical band intensity across samples demonstrates the suitability of β-tubulin as a stable loading control. At the bottom of these membranes the same sets of bands but at a higher intensity as they were used for further calculations are shown again (Fig. 1)

Figure 1.

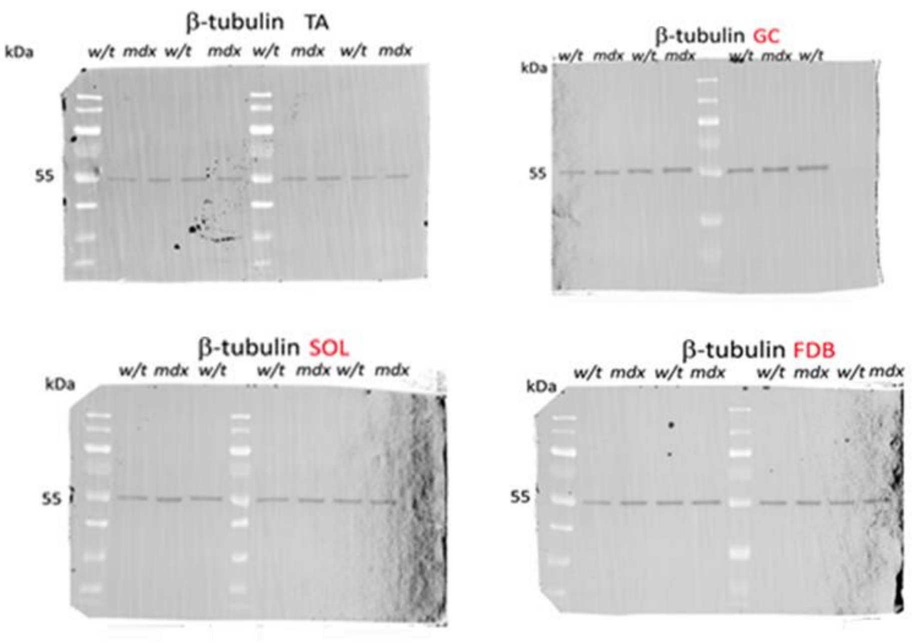

Figure 2.

Blots show the specific P2Y receptor (indicated) detected in lysates obtained from myoblasts derived from TA, GC, SOL, FDB isolated from three w/t and three mdx mice. Below each blot detecting specific P2Y receptor, the β-tubulin obtained as explained in Figure 1 are presented. These were used for the estimation of the relative amounts of the indicated P2Y receptors in myoblasts isolated from specific muscles.

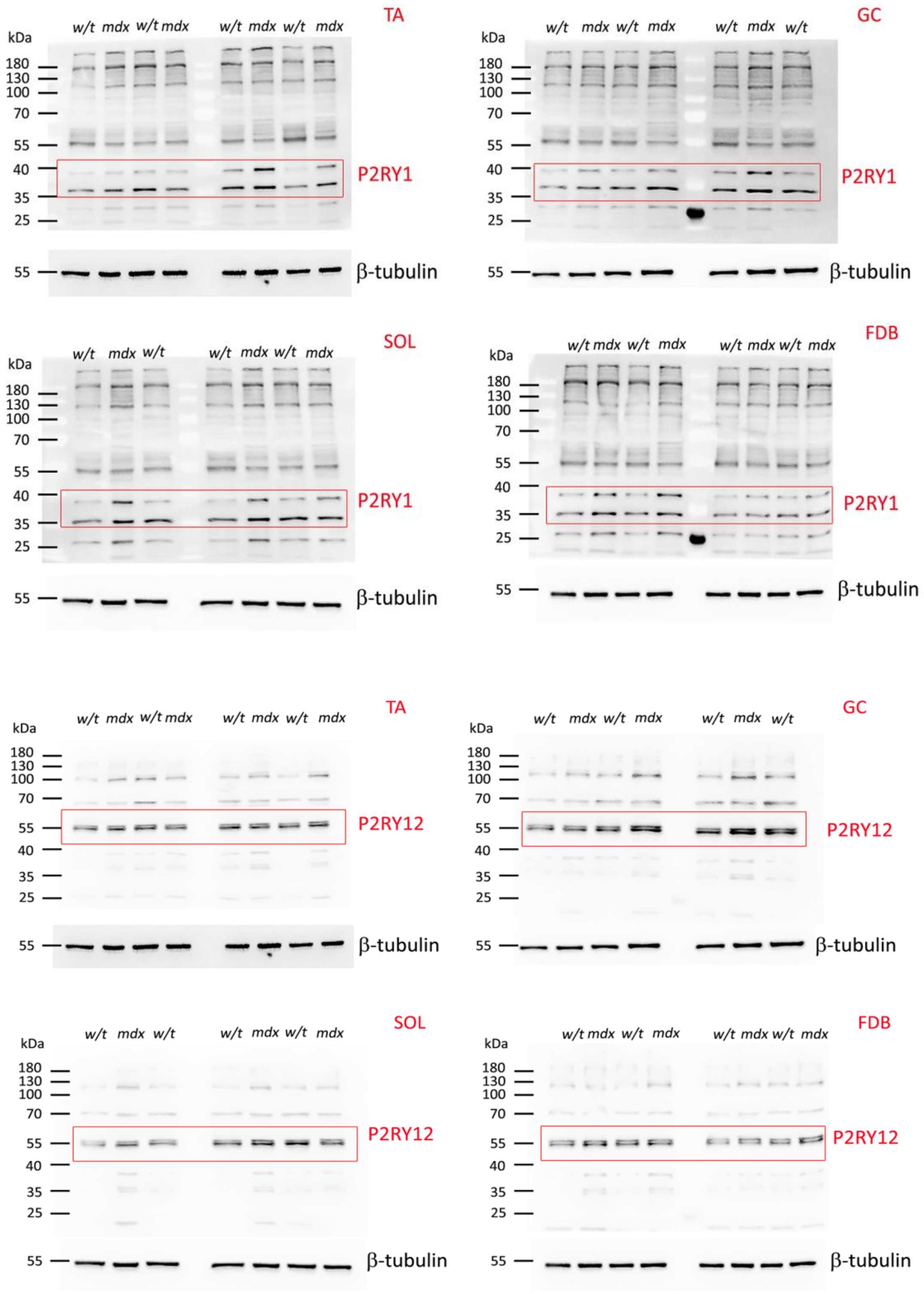
TA
GC
SOL
FDB
P2RY1
P2RY12
β-tubulin
kDa
180
130
100
70
55
40
35
25
w/t
mdx

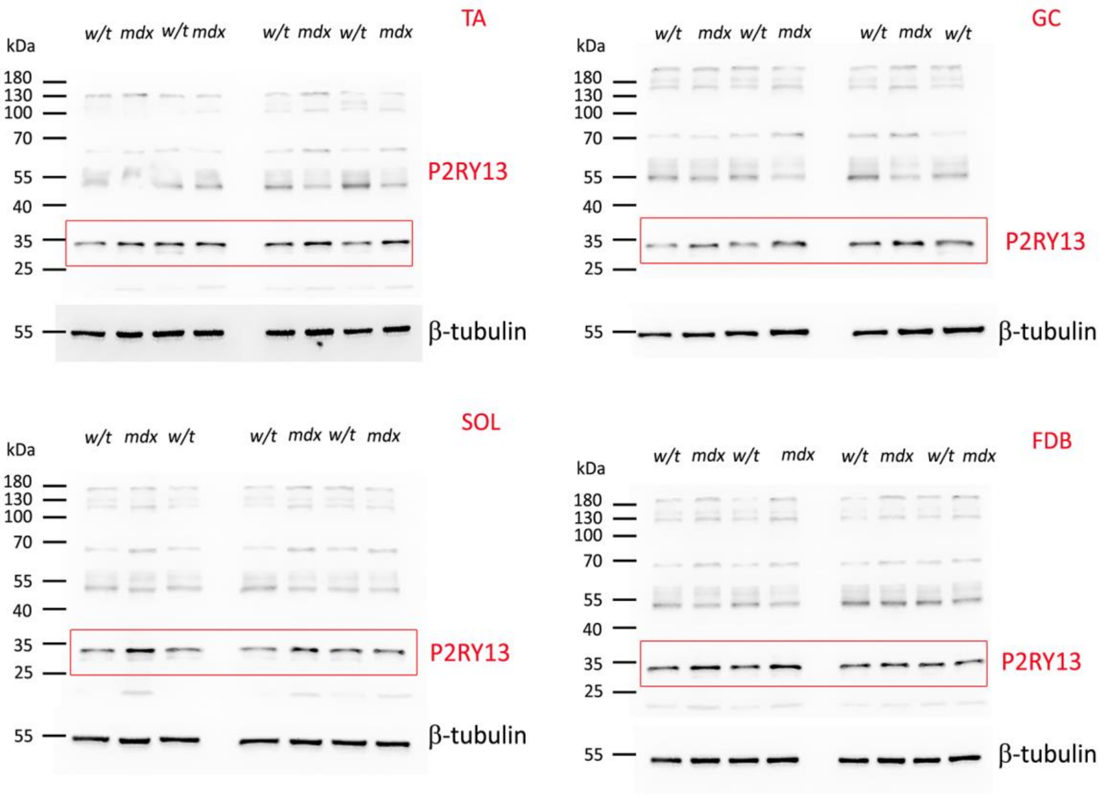

Figure 3

Western blots showing β-tubulin detection in samples used for analyses of P2RY2, P2RY4 and P2RY6.

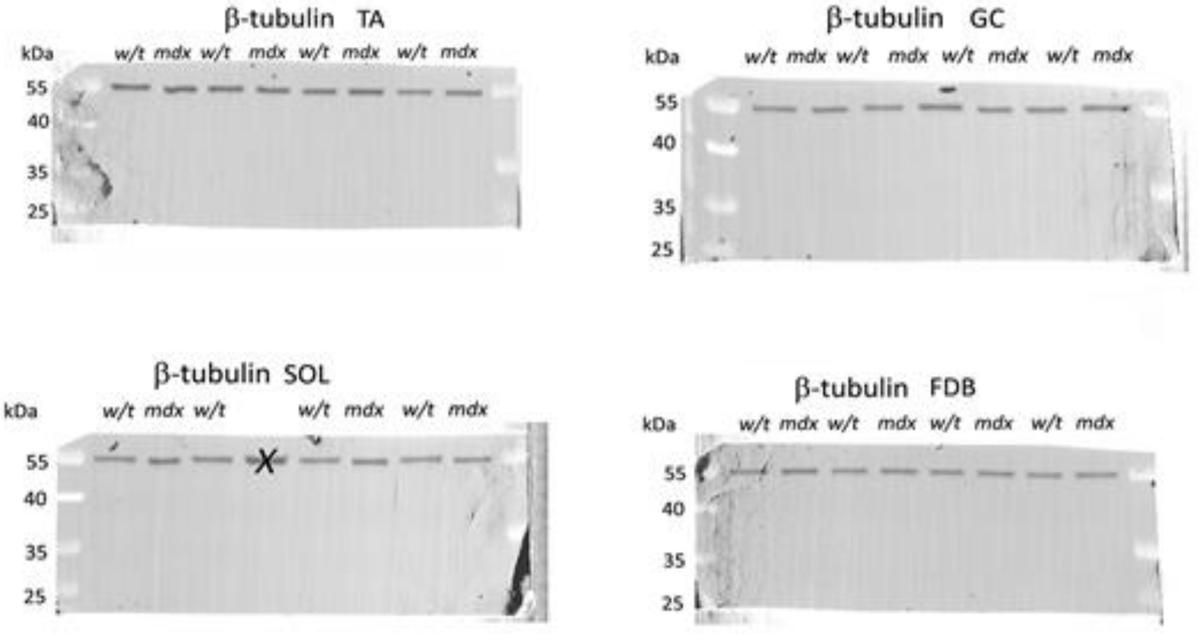

X Wrong sample loaded. This mistake was taken into consideration in further data analyses.

Figure 4

Blots show the specific P2Y receptor (indicated) detected in lysates obtained from myoblasts derived from TA, GC, SOL, FDB isolated from three w/t and three mdx mice. Below each blot detecting specific P2Y receptor, the β-tubulin bands are shown obtained as explained in Figure 3 are presented. These were used for the estimation of the relative amounts of the indicated P2Y receptors in myoblasts isolated from specific muscles.

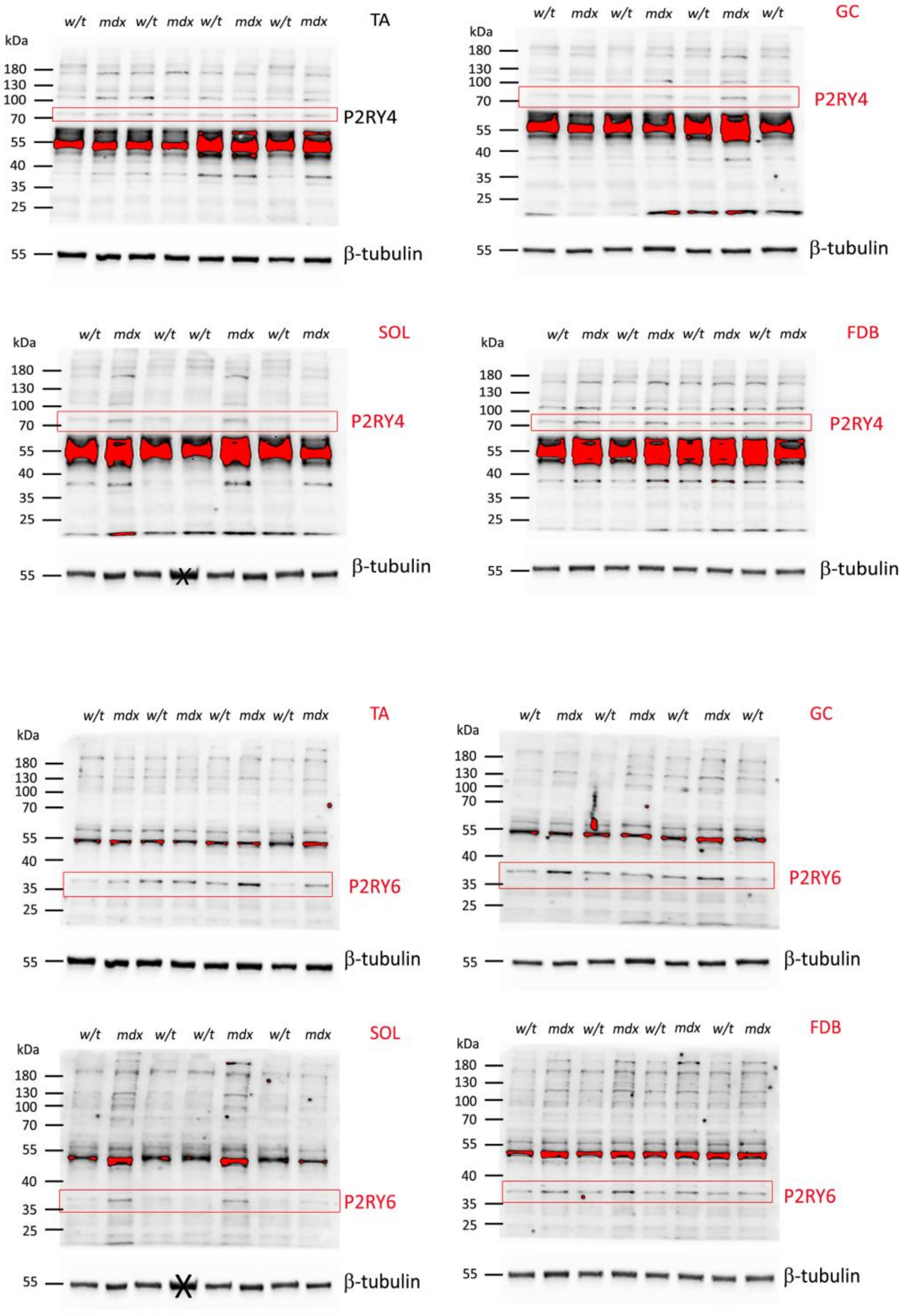

X Wrong sample loaded. This mistake was taken into consideration in further data analyses.

Western blots used for Calcium toolkit proteins detection

In this case, because of a large number of different proteins tested in the same sample, the membranes were cut into strips based on the molecular weights of proteins to be detected. It allowed avoiding membrane stripping, which can introduce additional errors. Moreover, using this approach, fewer animals could be used, as the same lysate was tested for multiple proteins.

β-tubulin was used as the loading control using the same approach described above and used to quantify all proteins of interest in samples from a specific muscle.

Each lane represents a protein sample of myoblasts isolated from a specific muscle from an individual mouse.

The left side shows the blot with protein mass markers. The middle is to show the raw data chemiluminescence merged with the mass markers visualised in the visible light (Fusion FX chemidoc). The right side shows blots with specific bands exposed to the intensity before signal saturation allowing comparisons of the expression levels.

TA

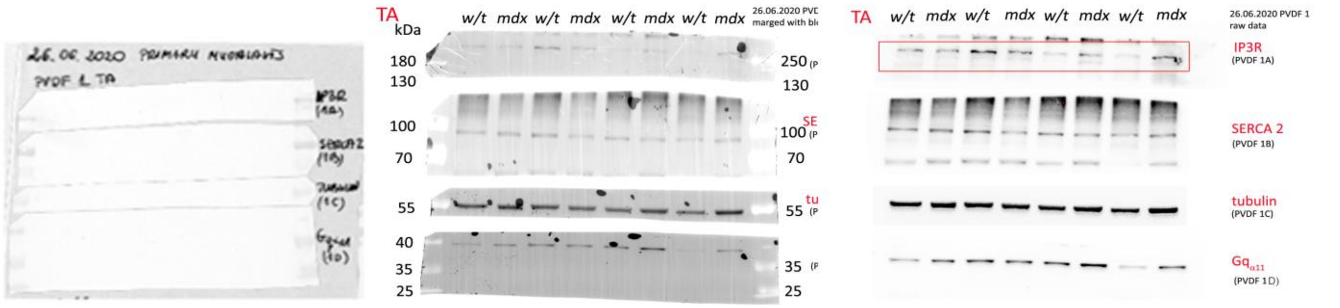

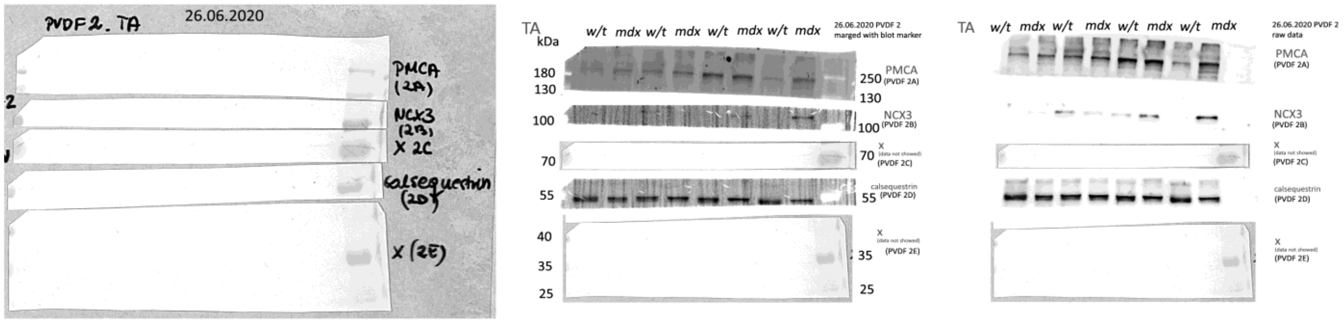

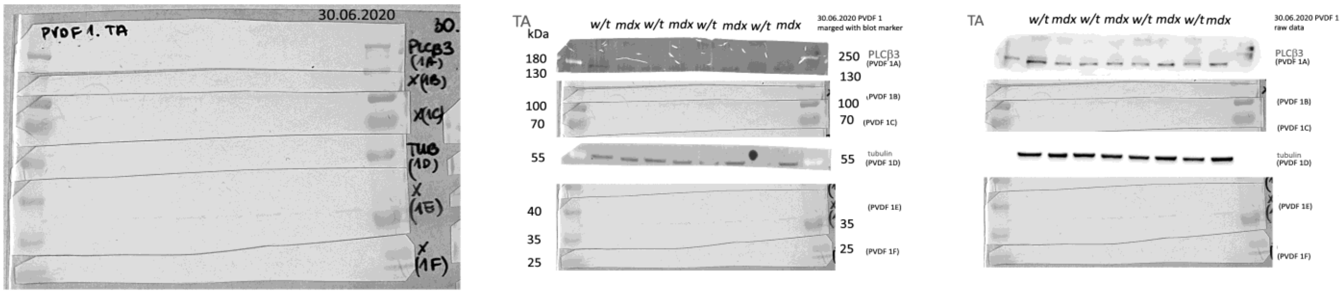

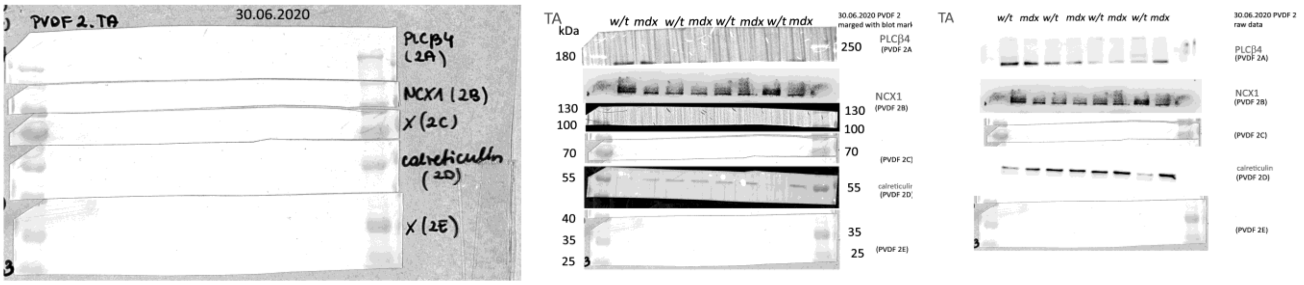

Empty strips correspond to proteins which were analyzed in those blots but are not relevant to this manuscript.

GC

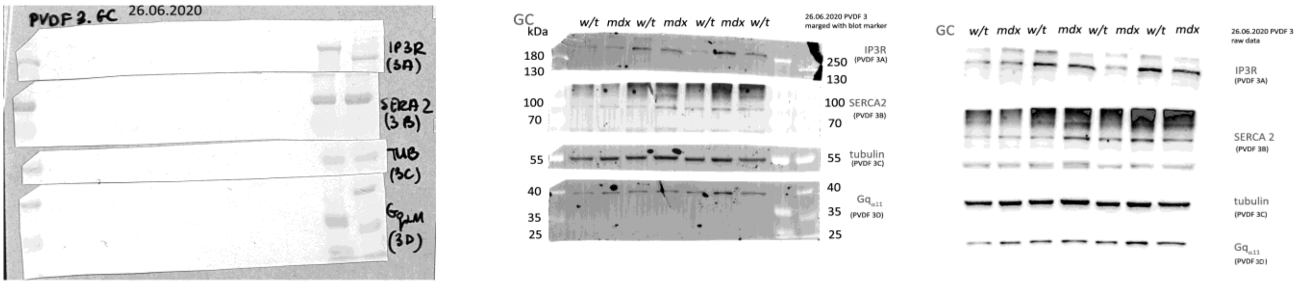

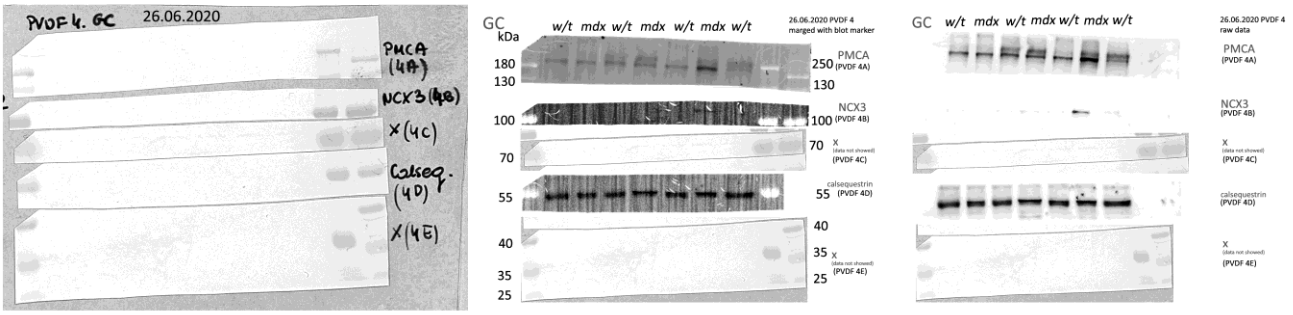

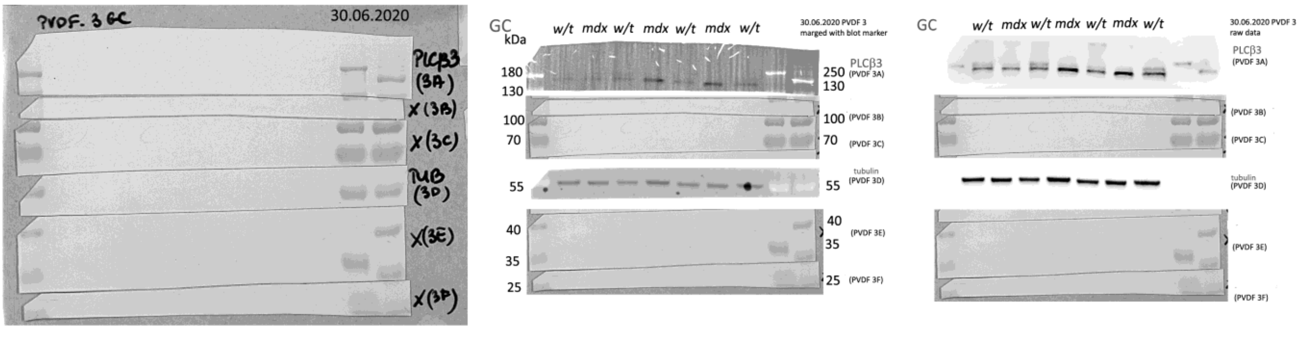

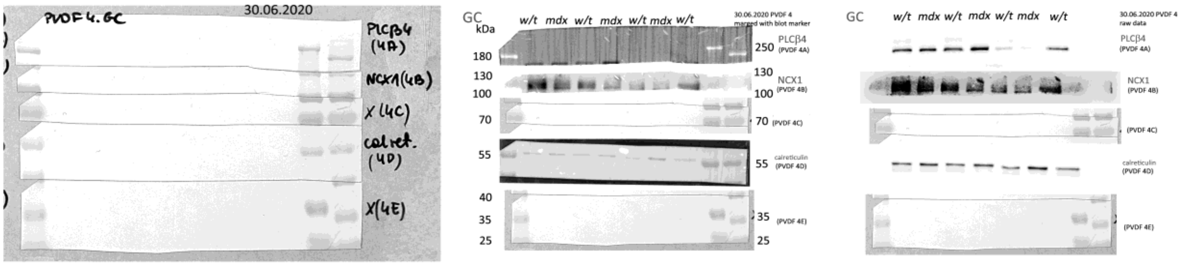

Empty strips correspond to proteins which were analyzed in those blots but are not relevant to this manuscript.

SOL

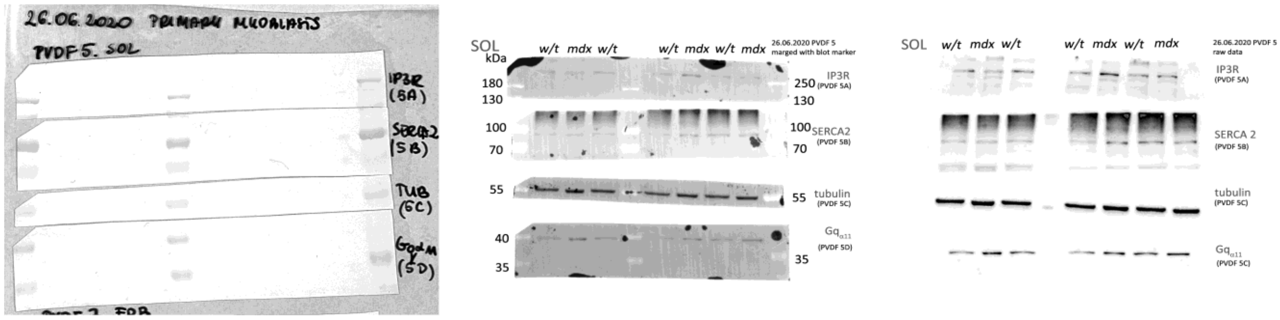

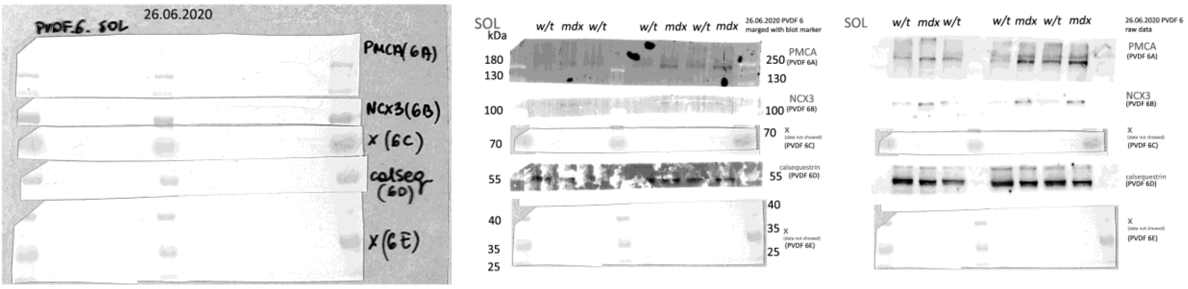

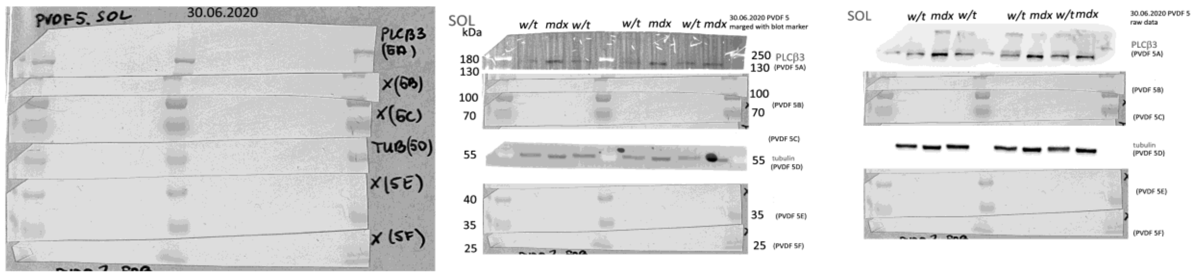

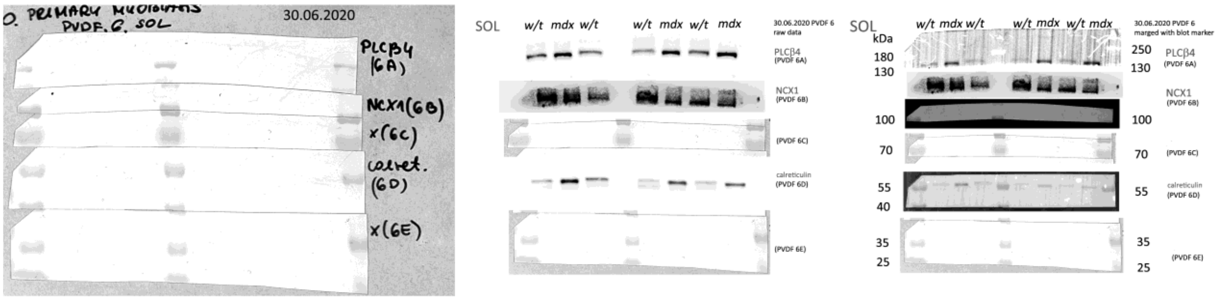

Empty strips correspond to proteins which were analyzed in those blots but are not relevant to this manuscript.

FDB

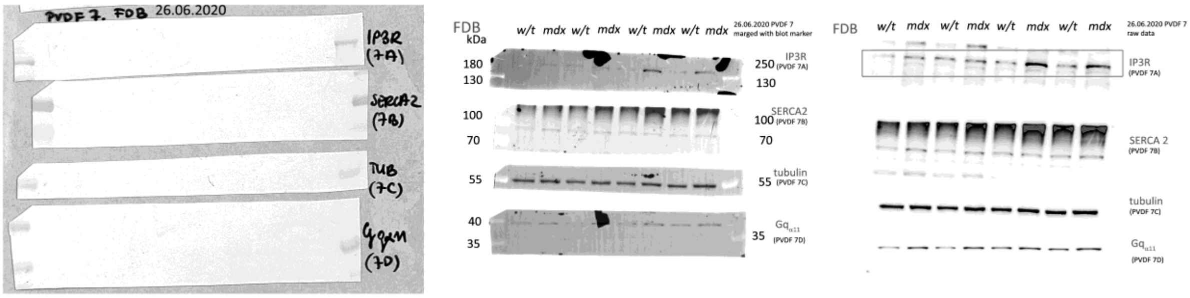

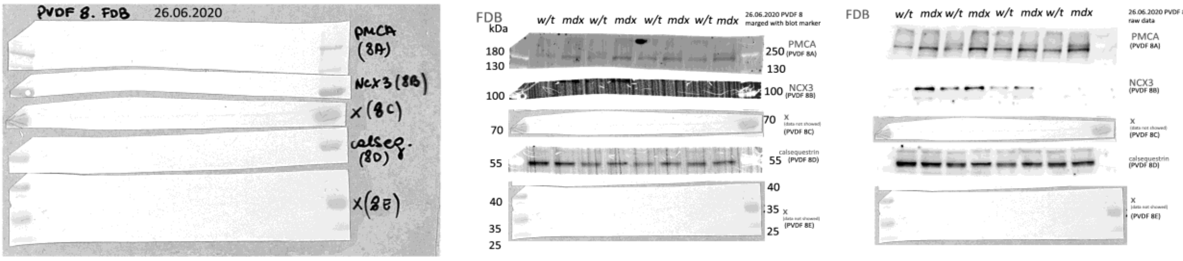

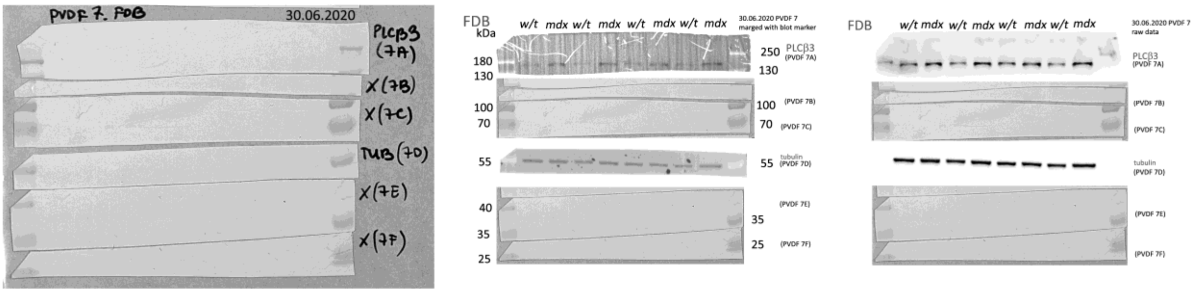

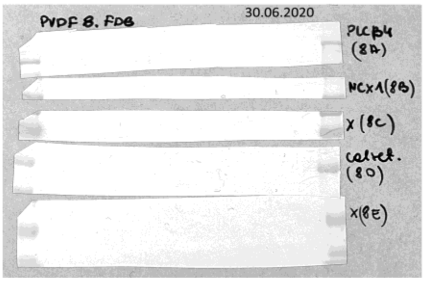

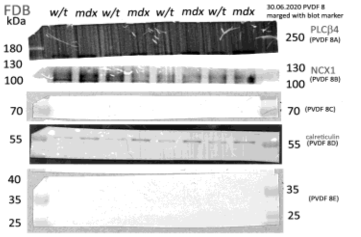

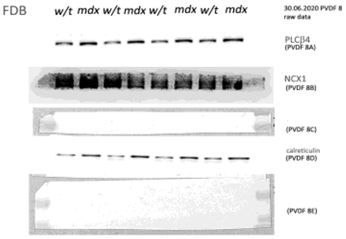

Empty strips correspond to proteins which were analyzed in those blots but are not relevant to this manuscript.
